# Evaluation of anakinra in the management of patients with COVID-19 infection: A randomized clinical trial

**DOI:** 10.3389/fmicb.2023.1098703

**Published:** 2023-01-26

**Authors:** Eman Zeyad I. Elmekaty, Aya Maklad, Rawan Abouelhassan, Waqar Munir, Mohamed Izham Mohamed Ibrahim, Arun Nair, Rim Alibrahim, Fatima Iqbal, Ahmad Al Bishawi, Alaaeldin Abdelmajid, Mohamed Aboukamar, Hamad Abdel Hadi, Mohammed Abu Khattab, Hussam Al Soub, Muna Al Maslamani

**Affiliations:** ^1^Communicable Diseases Center, Hamad Medical Corporation, Doha, Qatar; ^2^College of Pharmacy, QU Health, Qatar University, Doha, Qatar

**Keywords:** anakinra, COVID-19, interlukin-1 inhibitor, SARS-CoV-2, cytokine release syndrome, pneumonia

## Abstract

**Background:**

The global COVID-19 pandemic led to substantial clinical and economic outcomes with catastrophic consequences. While the majority of cases has mild to moderate disease, minority of patients progress into severe disease secondary to the stimulation of the immune response. The hyperinflammatory state contributes towards progression into multi-organ failure which necessitates suppressive therapy with variable outcomes. This study aims to explore the safety and efficacy of anakinra in COVID-19 patients with severe disease leading to cytokine release syndromes.

**Methods:**

In this open-label, multi-center, randomized clinical trial, patients with confirmed COVID-19 infection with evidence of respiratory distress and signs of cytokine release syndrome were randomized in 1:1 ratio to receive either standard of care (SOC) or anakinra (100 mg subcutaneously every 12 h for 3 days then 100 mg subcutaneously once daily for 4 days) in addition to SOC. The primary outcome was treatment success at day 14 as defined by the WHO clinical progression score of ≤3. Primary analysis was based upon intention-to-treat population, with value of *p* of <0.05.

**Results:**

Out 327 patients screened for eligibility, 80 patients were recruited for the study. The mean age was 49.9 years (SD = 11.7), with male predominance at 82.5% (*n* = 66). The primary outcome was not statistically different (87.5% (*n* = 35) in anakinra group vs. 92.5% (*n* = 37) in SOC group, *p* = 0.712; OR = 1.762 (95%CI: 0.39–7.93). The majority of reported adverse events were mild in severity and not related to the study treatment. Elevated aspartate aminotransferase was the only significant adverse event which was not associated with discontinuation of therapy.

**Conclusion:**

In patients with severe COVID-19 infection, the addition of anakinra to SOC treatment was safe but was not associated with significant improvement according to the WHO clinical progression scale. Further studies are warranted to explore patients’ subgroups characteristics that might benefit from administered therapy.

**Clinical Trial Registration:**

Trial registration at ClinicalTrials.gov, identifier: NCT04643678.

## Introduction

In March 2020 the World Health Organization (WHO), declared the severe acute respiratory syndrome coronavirus (SARS-CoV-2) and its related clinical entity of COVID-19 disease, as a global pandemic which continued to evolve leading to catastrophic consequences (Lai et al., 2020). As of November 2022, the total global cases exceeded 630 million with 6.6 million fatalities (WHO Coronavirus Disease (COVID-19) Dashboard, 2020). Although the majority of confirmed infections present with mild to moderate disease, clinical COVID-19 infection might progress towards severe sequela including multi-organs dysfunction such as acute respiratory distress syndrome (ARDS) with an estimated mortality of 3.4% (Guan et al., 2020; WHO, 2020).

The pathophysiology stems from activation of the immune response since the infection acts as an essential source for the activation of inflammatory cell lines such as macrophages and leukocytes, resulting in the release of pro-inflammatory cytokines primarily at local lungs tissues, followed by subsequent systemic responses. This process is thought to play a major role in the pathophysiology of subsequent organ dysfunction syndrome (Chen et al., 2020). As there is considerable biochemical overlap between cytokine storm syndrome and the hyper-inflammation status observed in patients with COVID-19 infection, emerged evidence from clinical trials directed toward screening patients with COVID-19 infection for signs of hyper-inflammation and consequently providing immunosuppressive management to reduce associated mortality (Mehta et al., 2020b; McGonagle et al., 2020).

Anakinra, is a recombinant non-glycosylated form of the human interleukin (IL)-1 receptor antagonist, which has demonstrated significant survival benefits in patients with macrophage activation syndrome (MAS) when compared to placebo in a subgroup analysis of patients with hyper-inflammation as a result of severe sepsis, reducing 28-day mortality to 35% as opposed to 65% (Shakoory et al., 2016). A large double blind randomized clinical trial encompassing almost 600 patients evaluated early Anakinra treatment of COVID-19 patients guided by the soluble urokinase plasminogen activator receptor (suPAR) levels, demonstrated significant combined reduction of 28 days mortality as well as shorter hospital stay (Kyriazopoulou et al., 2021b). Furthermore, numerous studies revealed that the administration of anakinra was associated with an acceptable safety profile where recent literature reported mild elevations in liver aminotransferases as the most common adverse event; however, such elevations were not clinically significant (Opal et al., 1997; Huet et al., 2020).

Therefore, numerous observational studies were conducted to investigate the efficacy and safety profile of anakinra in COVID-19 patients exhibiting features of severe disease encompassing cytokine storm syndrome highlighted promising results (Huet et al., 2020; Kooistra et al., 2020; Cavalli et al., 2021). However, such observational findings required extensive assessments in well-conducted randomized clinical trials to verify the reported outcomes (Cavalli et al., 2020; Langer-Gould et al., 2020). Later, few small randomized trials and meta-analyses were conducted to assess the benefit of anakinra in COVID-19 patients with inconclusive outcomes (Balkhair et al., 2021; Barkas et al., 2021; Declercq et al., 2021; Audemard-Verger et al., 2022; Kharazmi et al., 2022; Malık et al., 2022; Naveed et al., 2022). As in selected patients with severe COVID-19 pneumonia, anakinra has demonstrated beneficial outcomes, while it did not show a significant benefit in other patient groups (Balkhair et al., 2021; Barkas et al., 2021; Declercq et al., 2021; Audemard-Verger et al., 2022; Kharazmi et al., 2022; Malık et al., 2022; Naveed et al., 2022). The presented open-label randomized clinical trial aimed to explore the safety and efficacy of anakinra in addition to standard of care (SOC) compared to SOC alone in patients with confirmed COVID-19 infection with respiratory distress and evidence of cytokine release syndrome.

## Materials and methods

### Study design and setting

This multi-center study is an open-labeled prospective randomized clinical trial with parallel assignments. Patients were recruited from three clinical sites in Qatar: The Communicable Diseases Center (CDC), Hazm Mebaireek General Hospital (HMGH), and The Cuban Hospital (TCH) between October 30, 2020 till April 30, 2021. Eligible patients were randomized to receive either anakinra in addition to SOC compared to SOC alone. The trial protocol and the statistical analysis plan are available as Supplementary Material. The trial was approved by the Institutional Review Board at Hamad Medical Corporation (HMC) Medical Research Center (MRC-01-20-1,095) and was registered at ClinicalTrials.gov (NCT04643678).

### Participants

Hospitalized adults (age ≥ 18 years) were eligible for inclusion in the trial if they met the following eligibility criteria of severe disease according to local COVID-19 protocol guidelines: confirmed COVID-19 diagnosis by positive SARS-CoV2 Polymerase Chain Reaction (PCR) test and associated presence of respiratory distress [defined as: PaO2/FiO2 ≤ 300 mm Hg or respiratory Rate (RR) ≥24 breaths/min or SpO2 ≤ 94% at room air], with radiological evidence of pneumonia based on chest X-ray and/or computed tomography (CT) scan findings, signs of cytokine release syndrome [defined as any of the following at baseline: Ferritin >600 mcg/L at presentation or > 300 mcg/L with doubling within 24 h, LDH >250 IU/l, D-dimer >1 mg/L, CRP > 70 mg/L and rising since last 24 h with the absence of bacterial infection, Interleukin-6 level > 10 × UNL (reference range ≤ 7 pg/mL)], and a signed informed consent. The exclusion criteria included known serious allergic reactions including anaphylaxis to the study medication or any component of the product, active infectious diseases such as active bacterial infections, invasive fungal infections, Human Immunodeficiency Virus (HIV), Hepatitis B Virus (HBV) infection, Hepatitis C Virus (HCV) infection, active tuberculosis, patients on immunosuppressants or immunomodulatory drugs or had received any in the past 30 days, neutrophil count below 500 cells/microliter, platelets below 50,000/microliter, pregnant or breastfeeding females. A full list of eligibility criteria is provided in the Supplementary Material.

### Randomization and masking

All admissions with a laboratory-confirmed SARS-CoV-2 infection and on respiratory distress were screened for eligibility by the research team. Eligible patients were randomly assigned using simple randomization at an allocation ratio of 1:1 to receive either anakinra plus SOC therapy or SOC therapy alone. A random sequence of numbers was generated by an independent biostatistician using a computerized system. Only the principle investigator was responsible to allocate the participant serial number and inform the research team of the treatment allocation. Each participant was assigned an identification code corresponding to a study treatment group. The study was not blinded due to practical reasons and the differences in the dosage regimens and administrations between the study groups.

### Interventions and procedures

Patients in the intervention group received anakinra (KINERET®, 100 mg/0.67 mL in pre-filled syringe) 100 mg subcutaneous (SC) injection every 12 h for 3 days, then 100 mg SC once daily from day 4 to day 7. The total duration of anakinra was 7 days with a total of 10 doses. Patients with severe renal impairment (creatinine clearance <30 mL/min) had a 50% decrease in dose (dose administered every other day). Patients in the intervention group received anakinra in addition to standard of care therapy except for tocilizumab, due to the contraindication of administering both agents concomitantly. Patients in the comparator group received SOC therapy as per the local treatment guideline as approved by the Ministry of Public Health (MoPH) in Qatar at that time (see Supplementary Material). Apart from the study interventions and related laboratory and diagnostic assessments, patients were treated as part of standard care, with no co-medications, medical procedures, or diet restrictions. The study had pragmatic and adaptive nature, which mimics the usual clinical practice in a real-world setting and allows for changes in the management of COVID-19 patients and SOC therapy, as approved by the MoPH in the country.

After enrollment, a detailed review of the participant’s medical profile was conducted by the research team. Demographic, clinical, radiological, laboratory data were collected from each patient’s profile. The WHO Clinical Progression Scale (Marshall et al., 2020) was used to assess the patient’s clinical condition on a daily basis and was documented in the electronic data collection sheet. The worst score on each particular day was selected and recorded. The data collection sheet was validated independently by two investigators. The list of study procedures, timings, and follow-up is available in the Supplementary Material.

All adverse events (AEs) that occurred during the hospital stay and up to 28 days from randomization were reported. All AE were assessed for severity and graded using the Common Terminology Criteria for Adverse Events (CTCAE) version 5 (National Cancer Institute, 2017). A blinded assessor evaluated and validated the AE’s severity, seriousness, relatedness, and attribution using appropriate scales and/or definitions as per the HMC IRB Standard Operating Procedure guidelines.

Participants were followed up to 28 days from randomization. To ensure systemic and standardized follow-up of patients, they were contacted by telephone using a standardized telephone script on day 14 and day 28 to enable more accurate ascertainment of the study outcomes and adverse events occurring during and after hospital discharge.

### Outcomes

The primary outcome was treatment success on day 14, as defined by the 11-points WHO Clinical Progression scale of a score of ≤3 [Ambulatory mild disease: symptomatic, assistance needed]. The treatment success definition in the initial submission was WHO score of <6 [patient alive, not requiring invasive, non-invasive, or high flow oxygen therapy, vasopressors, dialysis or Extracorporeal membrane oxygenation (ECMO), however, as the majority of COVID-19 cases in Qatar were having mild–moderate disease with very low percentage of patients who require prolonged invasive ventilation or admission to ICU, the definition was revised in December 2020 and a protocol amendment was submitted and approved by the IRB.

Secondary outcomes included the duration of mechanical ventilation in ventilated patients up to 14 days, changes in the WHO Clinical Progression Score between day 1 and day 7, viral burden as measured by the change in SARS-CoV-2 PCR Cycle Threshold (CT) at day 7 and day 10–14, time to ICU admission up to 28 days, the incidence of adverse events up to 28 days, length of hospital stay up to 28 days, and all-cause mortality rate at hospital discharge or at 28 days, whichever occurred first.

### Statistical analysis

No published randomized trials evaluated the difference between the two study interventions at the time of study planning to appropriately estimate the mean difference and compute the sample size calculation. Therefore, a sample size of 80 patients was estimated for this RCT with parallel group design based on the hypothesized effect size from published observational studies with 80% power to detect a difference in the primary endpoint of 30%, with a two-sided type I error rate of 0.05 (alpha). Categorical data were expressed by frequency (percentage), while continuous values were expressed as mean ± SD or median and interquartile range (IQR), as appropriate. Data were tested for their normality. Kolmogorov–Smirnov test was carried to test for data normality. Continuous data were examined with the Mann–Whitney U or independent t-test and categorical data with the chi-square and Fisher’s Exact tests. Two-way repeated measure ANOVA analyzed the changes of data over time in the two treatment groups. Factors significant at univariate analysis (*p* < 0.10) were further assessed by the multi-variable binary regression analysis. All statistical analyses were carried out using the statistical package SPSS, version 27 (IBM Corp. Released 2020. IBM SPSS Statistics for Windows, Version 27.0. Armonk, NY: IBM Corp). The primary analysis was based on an intention-to-treat population, including all participants randomized.

### Ethics statement

The trial was approved by the Institutional Review Board at Hamad Medical Corporation (HMC) Medical Research Center (MRC-01-20-1,095) which abides by local and international ethical standards and was registered at ClinicalTrials.gov (NCT04643678). The trial was conducted in accordance with Good Clinical Practice guidelines and the principles of the declaration of Helsinki. An independent institutional review board and ethics committee approved the trial protocol and any subsequent amendments. The safety of the participants and the evaluation of the benefit–risk balance was overseen by an independent data safety monitoring committee. Written informed consent was obtained from all participants or legally authorized representatives before initiating any trial-related procedures. No additional administrative permissions were required to access the raw data. All data used in this study were fully anonymized before their use.

## Results

### Participants

**Figure 1:**
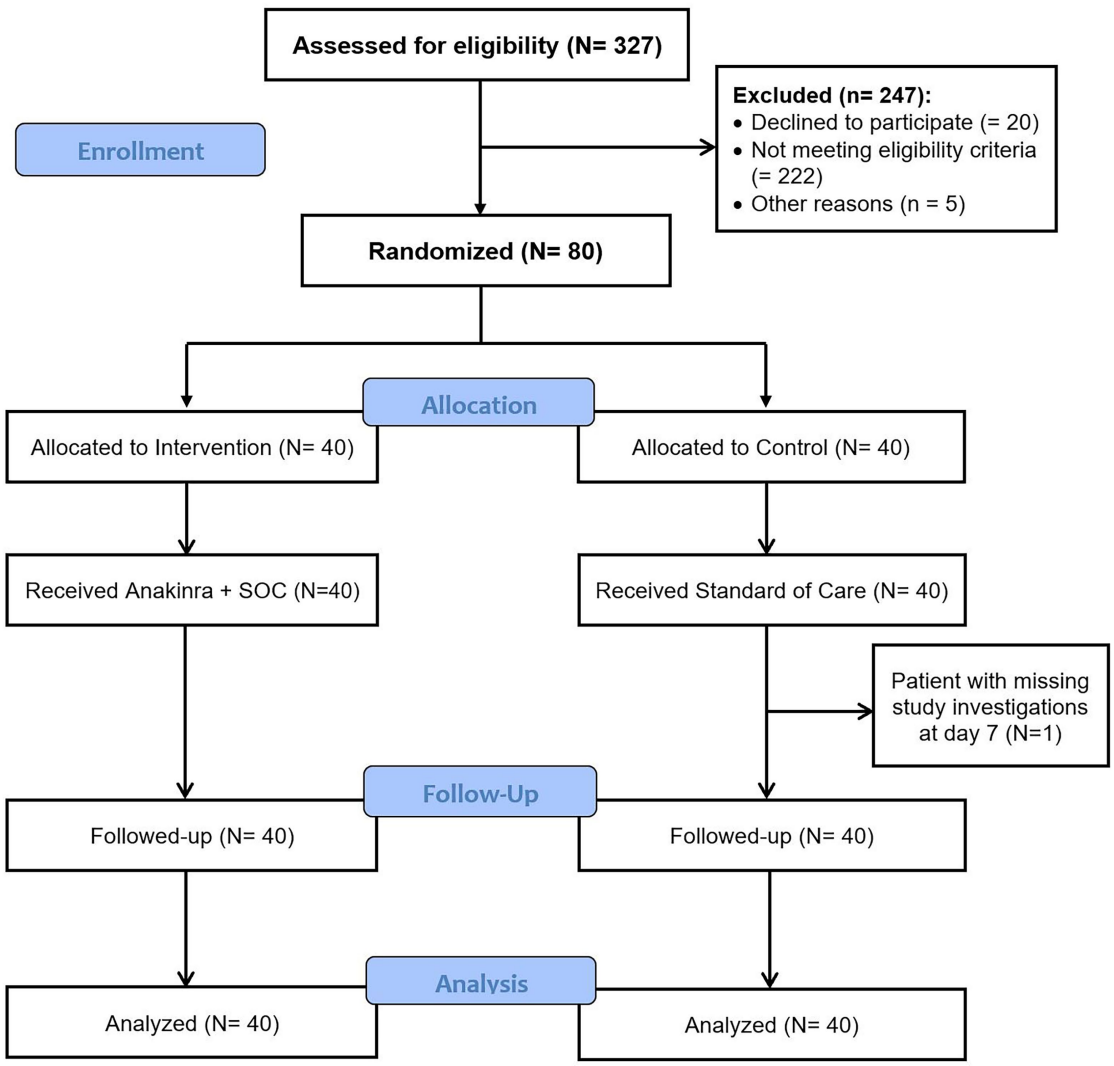
Flow diagram.

Between October 30, 2020 and February 28, 2021, 327 patients were screened for eligibility and 80 patients were enrolled and randomized to the two study groups: 40 patients in anakinra group and 40 patients in the SOC group. One patient in the SOC group was discharged from hospital against medical advice due to social reasons and did not complete the study investigations (Figure 1). All patients randomized were included in the intention to treat analysis. As enrolled patients were hospitalized during the treatment course, the compliance with study medication was 100% with no lost to follow-up.

The baseline characteristics of the patients were summarized in Table 1. The complete list of participants demographics, clinical data, and concomitant medications is available in the Supplementary Material.

**Table 1:** Baseline characteristics of the study population.

Characteristic	Overall (*n* = 80)	Anakinra (*n* = 40)	Standard of care (*n* = 40)
Mean Age, year (SD)	49.9 (11.7)	49.5 (12.2)	50.3 (11.4)
Gender, n (%)			
Male	66 (82.5)	32 (80)	34 (85)
Female	14 (17.5)	8 (20)	6 (15)
WHO Nationality Region, n (%)			
AFRO	1 (1.3)	1 (2.5)	0 (0)
PAHO	1 (1.3)	0 (0)	1 (2.5)
SEARO	34 (42.5)	13 (32.5)	21 (52.5)
EURO	2 (2.5)	0 (0)	2 (5.0)
EMRO	26 (32.5)	14 (35.0)	12 (30.0)
WPRO	16 (20.0)	12 (30.0)	4 (10.0)
Body mass index, median (IQR)	29.9 (27.3–32.9)	30.9 (7.0)	29.3 (5.2)
Smoking status, n (%)			
Non-smoker	72 (90.0)	36 (90.0)	36 (90.0)
Current smoker	0 (0)	0	0
Ex-smoker	8 (10.0)	4 (10.0)	4 (10.0)
Hospital, n (%)			
CDC	18 (22.5)	11 (27.5)	7 (17.5)
HMGH	44 (55.0)	20 (50.0)	24 (60.0)
TCH	18 (22.5)	9 (22.5)	9 (22.5)
Nurse unit, n (%)			
General ward	70 (87.5)	35 (87.5)	35 (87.5)
Intensive care unit	10 (12.5)	5 (12.5)	5 (12.5)
Has at least one comorbidity, n (%)	37 (46.3)	17 (42.5)	20 (50.0)
Myocardial infarction	7 (8.8)	2 (5.0)	5 (12.5)
Diabetes mellitus	35 (43.8)	17 (42.5)	18 (45.0)
Charlson comorbidity index (CCI), n (%)			
<2	72 (90.0)	37 (92.5)	35 (87.5)
≥2	8 (10.0)	3 (7.5)	5 (12.5)
Symptoms at baseline, n (%)			
Cough	75 (93.8)	36 (90.0)	39 (97.5)
Fever	69 (86.3)	34 (85.0)	35 (87.5)
Shortness of breath	59 (73.8)	29 (72.5)	30 (75.0)
Sore throat	12 (15.0)	4 (10.0)	8 (20.0)
Muscle/Joint pain	31 (38.8)	15 (37.5)	16 (40.0)
Chest pain	15 (18.8)	8 (20.0)	7 (17.5)
Headache	13 (16.3)	8 (20.0)	5 (12.5)
Weakness	14 (17.5)	5 (12.5)	9 (22.5)
Gastrointestinal side effect	25 (31.3)	9 (22.5)	16 (40.0)
Vital signs at baseline, mean (SD)			
Temperature (Max)	37.2 (0.6)	37.1 (0.7)	37.2 (0.6)
Systolic blood pressure (Min)	111.1 (13.0)	119.2 (19.2)	107.3 (16.9)
Diastolic blood pressure (Min)	63.5 (8.9)	73.2 (13.4)	56.5 (7.3)
Heart rate (Max)	88.4 (13.4)	95.4 (12.3)	99.5 (33.9)
Respiratory rate (Max)	22.9 (4.4)	29.2 (5.5)	32.0 (4.9)
O_2_ saturation % (Min)	93.4 (2.4)	93.0 (3.1)	92.3 (2.7)
PaO_2_/FiO_2_ (Min)	158.2 (42.1)	170.0 (57.1)	148.3 (26.0)
Labs at baseline, median (IQR)			
Ferritin	972.5 (695.1)	1087.3 (568.2)	990.5 (765.8)
Lactate dehydrogenase	424.1 (130.4)	450 (157.6)	349.5 (108.2)
D-dimer	0.6 (0.5)	0.4 (0.5)	0.5 (1.6)
C-reactive protein	81.3 (55.5)	41.0 (25.0)	68.9 (26.7)
Interlukin-6	23 (43.0)	25.0 (50.8)	22.0 (38.0)
WHO Score at baseline, median (IQR)	5 (0)	5.0 (0)	5.0 (0)
5 (%)	69 (86.3)	35 (87.5)	34 (85.0)
6 (%)	8 (10.0)	2 (5.0)	6 (15.0)
7 (%)	3 (3.7)	3 (7.5)	0
Bilateral abnormality at baseline x-ray	74 (92.5)	38 (95.0)	36 (90.0)
Concomitant medications			
Remdesivir (n, %)	67 (83.8)	36 (90.0)	31 (77.5)
Favipiravir (n, %)	42 (52.5)	18 (45.0)	24 (60.0)
Corticosteroids (n, %)	80 (100)	40 (100)	40 (100)
Convalescent plasma (n, %)	54 (67.5)	24 (60.0)	30 (75.0)
Azithromycin (n, %)	63 (78.8)	33 (82.5)	30 (75.0)
Ceftriaxone (n, %)	63 (78.8)	29 (72.5)	34 (85.0)
Anticoagulant use (n, %)	79 (98.8)	39 (97.5)	40 (100)

SD, standard deviation; IQR, interquartile range; CDC, communicable disease center, HMGH, Hazm Mebaireek General Hospital; TCH, The Cuban Hospital.

The change in vital signs and laboratory data over time (i.e., from baseline to day 14) were illustrated in Supplementary Figures S1–S36. No significant difference over time in the two treatment groups was noted except for the lymphocyte count (marginal; *p* = 0.046). Figure 2 demonstrates a significant change in WHO score (*p* ≤ 0.00) over 14 days. Test of significance between the two treatment groups denoting no significant difference between anakinra and SOC, and anakinra tended to delay improvement in WHO score.

**Figure 2:**
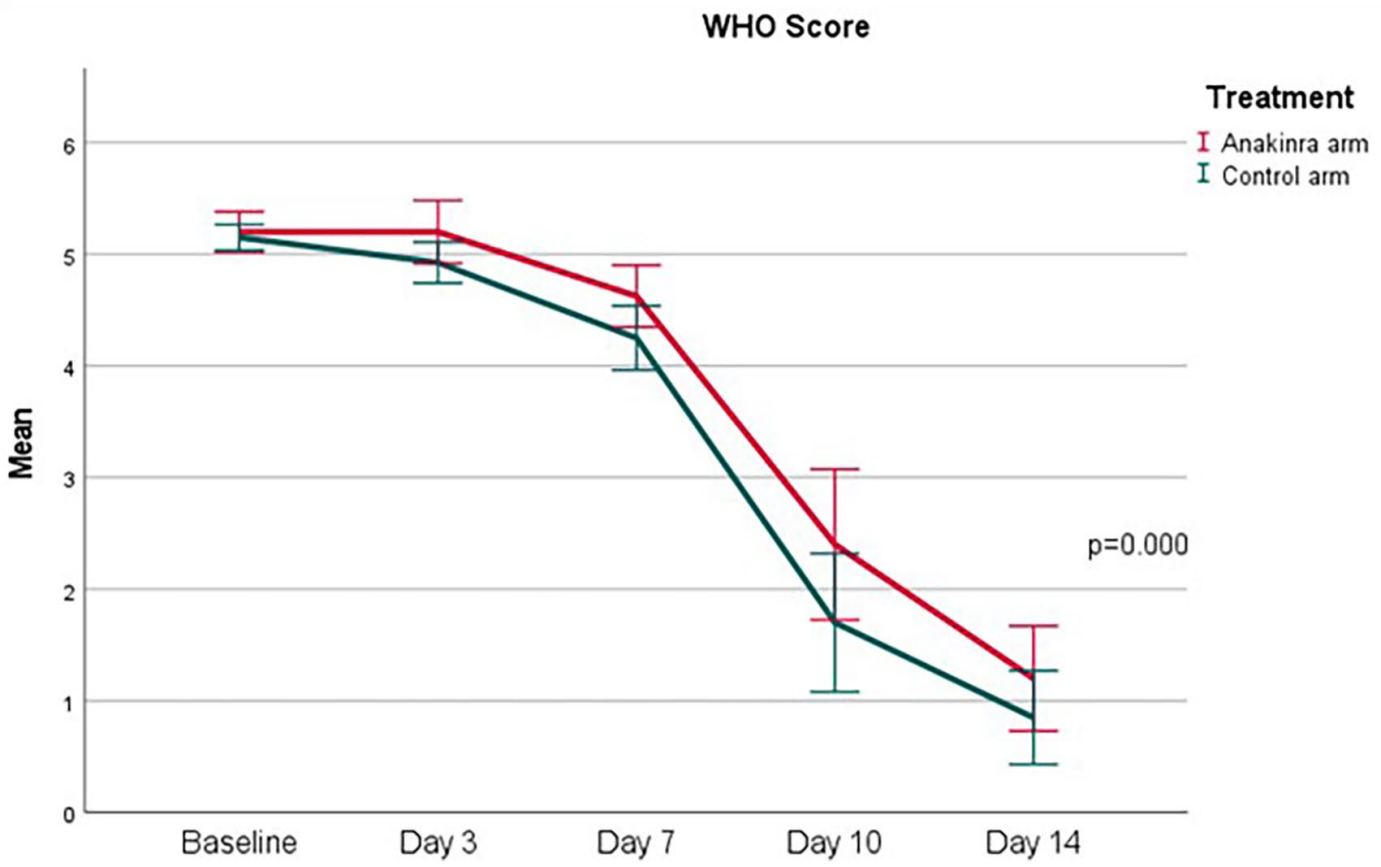
Change in the WHO clinical progression Score between day 1 and day 14.

### Outcomes

Table 2 below illustrates the results of the primary and secondary outcomes for the two treatment groups. The percentage of patients with treatment success at day 14 was not significantly different between the two groups (*p* = 0.712; OR = 1.762 (95% CI: 0.39–7.93). None of the secondary outcomes were found to be statistically significant. Three patients in anakinra group were intubated at baseline with a median duration of intubation of 6.5 days. One patient in anakinra group required intubation after randomization. None of the patients in standard of care group required intubation after randomization.

**Table 2:** Results of the primary and secondary outcomes.

Outcome	Overall (*n* = 80)	Anakinra (*n* = 40)	Standard of care (*n* = 40)	*p*-value
Primary outcome				
Percentage of patients with treatment success at day 14, n (%) [95% CI]	72 (90.0)	35 (87.5) [0.715–2.312]	37 (92.5) [0.290–1.835]	0.712
Secondary outcomes				
Duration of mechanical ventilation in ventilated patients up to day 14, median days (IQR) [95% CI]	6.50 (2.0)	6.50 (2.0) [4.73–7.77]	NA [na]	NA
Change in WHO score from baseline to day 7, median (IQR) [95% CI]	1 (0–1)	1 (1) [0.31–0.85]	1 (1) [0.58–1.2]	0.211
Change in viral burden (CT value) from baseline to day 7, median (IQR) [95% CI]	13.1 (7.8–23.2)	6.1 (14.9) [10.2–16.0]	9.8 (7.7) [13.8–19.9]	0.09
Change in viral burden (CT value) from baseline to day 10–14, median (IQR) [95% CI]	17.6 (8.5–23.9)	15.8 (16.0) [9.7–22.1]	17.6 (17.0) [9.4–21.1]	0.923
Time to ICU admission in non-ICU patients up to 28 days, median time (IQR) [95% CI]	6.0 (4.0–15.0)	7.5 (13.0) [0.2–3.3]	6.0 (15.3) [1.3–4.3]	1.00
Length of hospital Stay, median days (IQR) [95% CI]	10.0 (8.0–13.0)	10.0 (4) [10.4–13.8]	10.0 (4) [9.3–12.0]	0.064
All-cause mortality, n (%) [95% CI]	1 (1.25)	0 (0) [na]	1 (2.5) [na]	1.000

IQR, interquartile range; CT, cycle threshold; ICU, intensive care; CI, confidence interval.

The association between baseline characteristics and the primary outcome was not statistically significant between the two groups (refer to Supplementary Material).

### Safety outcomes

Two DSMB meetings were conducted as planned, in which the study’s progress, execution, results, and adverse event reports were reviewed by the committee members. At each meeting, the decision was made to proceed with the study accrual. A total of 377 adverse events were identified in the study. All patients had at least one adverse event, with an average of 4.7 events per patient. The majority of adverse events (61%) were mild in severity and did not cause harm to patients. Furthermore, 78% of the reported adverse events were judged by a blinded assessor to be not related to the study, 21% “possibly related,” and 1% “definitely related” to the study treatment. The most common adverse events were hypertension (65%), followed by increase in alanine aminotransferase, hypoalbuminemia, and sinus bradycardia (43.8% for the three events). Only increased aspartate aminotransferase level was found to be significantly higher in the anakinra group compared to the SOC group (35% vs. 15%), respectively (*p* = 0.039). Four serious adverse events were reported in the study, two in the anakinra group and two in the SOC group. To elaborate, one patient in the SOC died 23 days after randomization due to worsening of his pre-existing medical condition, while the second patient had a prolonged hospital stay due to diarrhea, in which both were judged to be unrelated to study interventions. In the anakinra group, both patients were re-admitted to the hospital for 3–6 days after 2 weeks of randomization due to suspected respiratory tract infections, which were judged to be possibly related to the study interventions. Table 3 summarizes the common adverse events reported in the study.

**Table 3:** Reported adverse events for study groups.

Adverse events, n (%)	Overall (*n* = 80)	Anakinra (*n* = 40)	Standard of care (*n* = 40)	*P*-value
Incidence of at least one AE	80 (100)	40 (50)	40 (50)	NA
Average number of AE per patient	4.7	4.7	4.72	0.034
Serious AE	4 (5)	2 (2.5)	2 (2.5)	NA
Number of adverse events	377/377 (100)	201/377 (53)	176/377 (47)	NA
Severe AE (grade 4–5)	1/377 (0)	0/377 (0)	1/377 (0)	NA
Mild–Moderate AE (grade 1–3)	376/ 377 (100)	201/377 (53)	175/377 (46)	0.034
Relatedness to study intervention				
Not related	294/377 (78)	154/377 (41)	140/377 (37)	0.863
Possibility related	79/377 (21)	44/377 (12)	35/377 (9)	0.534
Probably related	2/377 (1)	1/377 (0)	1/377 (0)	NA
Definitely related	2/377 (1)	2/377 (1)	0/377 (0)	NA
Common AE (incidence >5%)				
Hypertension	52 (65.0)	27 (67.5)	25 (62.5)	0.639
Alanine aminotransferase increased	35 (43.8)	19 (47.5)	16 (40.0)	0.499
Hypoalbuminemia	35 (43.8)	18 (45.0)	17 (42.5)	0.822
Sinus bradycardia	35 (43.8)	16 (40.0)	19 (47.5)	0.499
Hypotension	32 (40.0)	19 (47.5)	13 (32.5)	0.171
Anemia	21 (26.3)	8 (20.0)	13 (32.5)	0.204
Aspartate aminotransferase increased	20 (25.0)	14 (35.0)	6 (15.0)	0.039
Hyponatremia	20 (25.0)	11 (27.5)	9 (22.5)	0.606
Hyperglycemia	17 (21.3)	9 (22.5)	8 (20.0)	1
Lymphocyte count decreased	15 (18.8)	8 (20.0)	7 (17.5)	0.775
ECG QT corrected interval prolonged	9 (11.3)	5 (12.5)	4 (10.0)	1
Alkaline phosphatase increased	8 (10.0)	5 (12.5)	3 (7.5)	0.712
Diarrhea	8 (10.0)	3 (7.5)	5 (12.5)	0.712
Hyperkalemia	7 (8.8)	5 (12.5)	2 (5.0)	0.432
Hypokalemia	7 (8.8)	3 (7.5)	4 (10.0)	1
Sinus tachycardia	6 (7.5)	4 (10.0)	2 (5.0)	0.675
Lymphocyte count increased	4 (5.2)	2 (5.0)	2 (5.0)	1.000
Creatinine increased	4 (5.2)	2 (5.0)	2 (5.0)	1.000

AE, adverse event; ECG, electrocardiogram.

## Discussion

The unprecedented pandemic caught the unprepared healthcare settings across the globe leading to catastrophic clinical and economic consequences (Kaye et al., 2021). Despite the unwitnessed extensive research and development during the pandemic to examine the pathology of disease towards the discovery of appropriate therapy, modest achievements were accomplished (Sachs et al., 2022).

Despite the plausible concept of repurposing immune modulating therapy, this multi-center, open-labelled, randomized clinical trial found that anakinra administered in addition to SOC did not achieve intended efficacy outcomes when compared to SOC however it was safe and well tolerated with no significant associated adverse events. The use of anakinra was also not associated with a significant change in the WHO clinical progression score or reducing viral burden at 14 days when compared to standard care. The length of stay and the time to ICU admission for non-ICU patients did not differ between the two groups. The overall mortality rate was low in this trial (1.25%) as only one death was reported in the SOC group.

The main findings in our trial are consistent with that of the randomized clinical trial by the CORIMUNO collaborative group from France (CORIMUNO, 2021). The CORIMUNO-ANA-1 trial was an open-label, multi-center, randomized controlled trial of anakinra administered intravenously (IV) for 5 days compared to usual care to assess its survival benefits and the need for mechanical ventilation at day 4. Similar to our population, the CORIMUNO trial included patients with a WHO clinical progression score of ≥ 5. The two primary outcomes were not significant, and the need for mechanical or non-invasive ventilation at day 14 was also not different in both groups. Overall mortality at days 28 and 90 did not differ between the groups, and as a result the trial was prematurely stopped for futility. Nevertheless, other contrasting studies such as SAVE-MORE, a randomized clinical trial which recruited almost 600 patients advocated early therapy with anakinra guided by suPAR levels, concluded favorable outcomes both on mortality and duration of hospital stay (Kyriazopoulou et al., 2021b).

Anakinra is approved by the Food and Drug Agency (FDA) for the treatment of rheumatoid arthritis, neonatal-onset multi-system inflammatory disease, and deficiency of IL-1 receptor antagonist. It has also been utilized in various other clinical situations, most notably its off-label use in the treatment of hemophagocytic lympho-histiocytosis and cytokine storms (Rajasekaran et al., 2014; Mehta et al., 2020a). The hyperinflammation seen in patients with COVID-19 pneumonia is associated with severe disease presentation and is often identifiable by elevation of common inflammatory markers such CRP, IL-6, and procalcitonin, as well as other cytokines (Zeng et al., 2020). In addition, earlier studies suggested a significant role of raised cytokines, including IL-1, in severe COVID-19 pneumonia, which hypothesized a potential role of IL-1 inhibitors in patients with COVID-19 pneumonia (Mehta et al., 2020a,b).

It worth highlighting that the COVID-19 pandemic explored the pathogen-host immune response interactions like never before. Despite the extensive research in the subject during the pandemic, detailed steps in the pathophysiology of the exact role or innate or adaptive immunity including cytokines release such as IL-1 are not fully determined (Van de Veerdonk et al., 2022). Nevertheless, the essential role of the immune responses towards acute infection or vaccination was clearly established including their unregulated production leading to the development of cytokine release syndrome hence necessitating immune modulating therapy (Hosseini et al., 2020; Triggle et al., 2021)^.^ Most likely this variation involves both underlying host and pathogen factors accentuated by the context of the environment. Similarly, the exact role of the host naturally occurring interleukin-1 family of cytokines as well as related monoclonal competitive inhibitors such as anakinra or canakinumab towards SARS-Cov-2 virus acute infection, secondary immune response including vaccination is not fully determined. The contentious area during the pandemic was the clinical benefits and progress including relapses of patients with auto-immune or auto-inflammatory conditions such as rheumatoid arthritis treated with IL-1 inhibitors such as anakinra who subsequently develop acute COVID-19 infections including variants of concerns or being eligible for vaccination because of high risk status. Despite the documented lower efficacy results of vaccination and unanswered potential relapses, recommendations inclined for benefits that outweighed potential risks (Atagündüz et al., 2021).

Several case series and observational studies suggested the potential clinical improvement and mortality benefit of anakinra use in COVID-19 pneumonia (Aouba et al., 2020; Dimopoulos et al., 2020; Navarro-Millán et al., 2020; The RECOVERY Collaborative Group, 2020; Somagutta et al., 2021). A recent meta-analysis of 15 observational and interventional studies with a total of 3,530 patients suggested that there is an overall mortality benefit with the use of anakinra when compared to usual care (Somagutta et al., 2021). However, these studies were limited by the lack of randomization and the potential for selection and confounding bias, as well as the significant heterogeneity observed in the meta-analysis outcomes.

Unfortunately, the mortality benefit observed in the previous observational studies was not observed in our trial or the CORIMUNO-ANA-1 trial (CORIMUNO, 2021). Despite including patients with severe COVID-19 pneumonia, this trial reported only one patient death (1.25%) in the SOC group. On the other hand, the CORIMUNO-ANA-1 trial reported an overall mortality rate of 28% in each group. The advanced SOC may partly explain the low mortality rate in this trial compared to the CORIMUNO-ANA-1 trial. To explain, in the three sites, SOC for all patients included administration of dexamethasone and remdesivir as per the national treatment guidelines. However, none of the CORIMUNO-ANA-1 trial patients received antivirals like remdesivir or favipravir, and dexamethasone was not established as part of routine care for moderate to severe pneumonia by then. Dexamethasone in the RECOVERY trial showed mortality benefit and, eventually, became standard of care globally for treating severe COVID-19 (The RECOVERY Collaborative Group, 2020). The Infectious Disease Society of America treatment guidelines recommend using remdesivir in patients requiring supplemental oxygen and in patients with moderate to severe COVID-19 pneumonia, given its proven clinical benefits in this population (Bhimraj et al., 2020). Furthermore, in an observational cohort study assessing the efficacy and safety of the association of anakinra and steroid use in severe COVID-19 patients, the risk of death was significantly lower in treated patients compared to controls (Bozzi et al., 2021).

There is an increased interest to prevent severe respiratory failure and the need for intubation and mechanical ventilation in patients presenting with COVID-19 pneumonia. Small case series from the United States initially showed that IV administration of anakinra prevented the development of severe acute hypoxic respiratory failure (Navarro-Millán et al., 2020). Another meta-analysis of seven studies (including the CORIMUNO ANA-1 trial), with a total of 740 patients receiving anakinra, showed that anakinra reduced the need for invasive mechanical ventilation (Somagutta et al., 2021). The considerable heterogeneity in these studies as well as the predominantly retrospective and observational nature of those studies, warranted the confirmation of these findings in a randomized clinical trial. However, no significant benefit was observed by either this trial or the CORIMUNO-ANA-1 trial with regards to the rates of invasive mechanical ventilation.

The preferred core outcomes for clinical studies as defined by the WHO working group (Marshall et al., 2020) were used in this trial. Cycling Threshold is used as a marker of viral load, as it is inversely related to the viral load and provides an indirect method of quantifying the copy number of viral RNA (Rao et al., 2020). Anakinra did not affect the viral burden when compared to the SOC group in this study. Furthermore, the WHO clinical progression scale was chosen as an objective outcome measure to reduce the variability and heterogeneity seen in the previous studies and allow comparisons with future studies. No significant changes were reported in the baseline of the WHO clinical progression score or the seven-days score between the two groups.

Overall, a higher prevalence of adverse events was reported in this trial as compared to other studies, with every patient experiencing at least one adverse event. The most commonly reported adverse events were hypertension, raised liver transaminases, and hypoalbuminemia. Only AST elevation was significantly higher in the anakinra group, but the elevation was transient and corrected following discontinuation of therapy. Although AST elevation is one of the possible adverse effects of anakinra in the intervention group, the possibility that the reported increase in this trial might be due to other multiple concomitants administered medications. Overall, the incidence of severe adverse events did not exceed 5%. The high prevalence of adverse events in our trial may partially be attributed to the robust data collection and the additional concomitant use of antivirals as part of SOC therapy compared to SOC in other studies.

Despite our negative outcome of the beneficial efficacy of anakinra in patients with severe COVID-19 disease, this must be examined against available evidence. While some systematic reviews and meta-analysis concluded definite beneficial outcomes for Anakinra in term of reducing hospital stay and mortality without jeopardizing progression towards adverse events, others highlighted insufficient evidence pointing towards heterogeneity of included patients’ characteristics (Kyriazopoulou et al., 2021a; Somagutta et al., 2021; Davidson et al., 2022). Nevertheless, since the immunological role of cytokines including IL-1 is clearly demonstrated in severe and critical COVID-19 disease, it is plausible to postulate that immune suppressive therapy including treatment with anakinra is beneficial is such settings provided it entails the careful selection of targeted cohort (Davidson et al., 2022).

Furthermore, there are potential contributary factors that should be taken into consideration. From the review of the role of anakinra during the management of patients with COVID-19 disease, there are no clear evidence of the optimal timing of intervention, route of administration, exact relevant biomarkers or ideal dose or duration (Khani et al., 2022)^.^ From previous studies, there is increased awareness of the concept of the possible narrow therapeutic window to treat cytokine storms with cytokine inhibitors as well as the right time to initiate IL-1 inhibitors (Cavalli and Dagna, 2021). There is a need to define if certain inflammatory markers or other biomarkers such as SuPAR to guide the right timing for the use of anakinra. This has been investigated in a double-blinded placebo-controlled randomized trial (SAVE-MORE) assessing the effects of the biomarker-guided therapy demonstrating beneficial outcomes (Kyriazopoulou et al., 2021b). Similarly, another non-randomized study, where ferritin was used as a biomarker to guide therapy, showed significantly improved clinical outcomes, including reduced mortality rates and progression to severe COVID-19 by early administration of therapy either with anakinra alone or in combination with steroids (Dalekos et al., 2021). Due to the unavailability of kit used to measure the soluble urokinase plasminogen activator receptor (suPAR) serum levels in the facility, this biomarker was not measured in this trial.

It is worth highlighting that the study was conducted in late 2020 when the major circulating SARS-COv2 variant was the wild-type with its known morbidity and mortality before the evolution of other variants of concern in the country (Butt et al., 2022)^.^ Additionally, vaccination was introduced to the public in early 2021 so we assume, little implications of viral characteristics or enhanced immune status on the outcomes. Nevertheless, we acknowledge that the trial has some limitations. First, a placebo was not used in this trial due to the nature of the pandemic and the urgency to answer the clinical question. In addition, there is a potential for bias due to the open-label nature of the trial. However, this was minimized by choosing objective outcomes and blinding the outcome assessors. Finally, the overall sample size was small, especially for patients with severe disease or mechanical ventilation; hence, subgroups analysis in this population was not feasible.

On the other hand, this trial has several strengths. This study is a multi-center clinical randomized trial conducted on COVID-19 patients after establishing a better SOC (steroids and remdesivir). Moreover, it was a multi-center trial involving multiple ethnic groups, contributing to the study results’ higher generalizability. The WHO’s recommended outcomes for clinical studies were used; those included the WHO clinical progression score, viral burden as measured by the cyclic threshold, and the overall mortality, which facilitate the comparison of this trial’s results to other similar trials. Finally, no significant loss of follow-up rate was reported in this study.

## Conclusion

In patients with severe COVID-19 pneumonia requiring oxygen therapy, the addition of anakinra to the SOC treatment was safe but not associated with significant improvement in the WHO clinical progression scale. Therefore, there is a need for further research to determine subgroups of COVID-19 patients who might benefit the most by the timely administration of anakinra.

## Data availability statement

The datasets presented in this article are not readily available because the datasets used and analyzed during the current study are available from the corresponding author on reasonable request. Requests to access the datasets should be directed to eelmekaty@hamad.qa.

## Ethics statement

The trial was reviewed and approved by the Institutional Review Board at Hamad Medical Corporation (HMC) Medical Research Center (MRC-01-20-1095). The safety of the participants and the evaluation of the risk-benefit balance was overseen by an independent data safety monitoring committee. The patients provided their written informed consent to participate in this study.

## Author contributions

EE: conceptualization and methodology. MA, WM, RAl, and EE: data validation. MI: formal analysis and visualization. WM, AB, AN, AM, RAb, FI, MK, RAl, MA, and AA: investigations. HS, MK, and MM: resources. AM, RAb, and EE: data curation. AB, AN, MI, and FI: writing – original draft. AM, RAb, MK, EE, HS, and AA: writing – review and editing. HS and MM: supervision. All authors contributed to the article and approved the submitted version.

## Funding

This study was funded by the Medical Research Center at Hamad Medical Corporation, Qatar (MRC-01-20-1095).

## Conflict of interest

All authors declare that the research was conducted in the absence of any commercial or financial relationships that could be construed as a potential conflict of interest.

## Publisher’s note

All claims expressed in this article are solely those of the authors and do not necessarily represent those of their affiliated organizations, or those of the publisher, the editors and the reviewers. Any product that may be evaluated in this article, or claim that may be made by its manufacturer, is not guaranteed or endorsed by the publisher.
