# Supplementary material for: Evaluation of anakinra in the management of patients with COVID-19 infection: A randomized clinical trial
Source: Front Microbiol. 2023 Jan 26;14:1098703. doi: 10.3389/fmicb.2023.1098703 (PMC9910697; doi:10.3389/fmicb.2023.1098703)
Supplement: Supplementary file 1 [file Data_Sheet_1.docx]

**Supplementary Materials**

**Efficacy of Anakinra in the Management of Patients with COVID-19 Infection: A Randomized Clinical Trial**

This appendix has been provided by the authors to give readers additional information about their work.

**Table of Content**

1. .Completed list of the eligibility criteria …………………………………………………… 3

2. Baseline characteristics of the participants …………………………………………..…… 4

3. Laboratory and vital signs results over time ……………………………………………… 10

4. Association between baseline characteristics and primary outcome table ……..….……… 29

5. Completed list of adverse drug event for the trial population ……….………………….… 31

**Supplementary Figure 1. Completed list of eligibility criteria**

| Inclusion Criteria | Exclusion Criteria |
| --- | --- |
| Hospitalized adults (age ≥ 18yrs) male or female | **Known serious allergic reactions including anaphylaxis to the study medication, or any component of the product** |
|  |  |
| Confirmed COVID-19 diagnosis by positive SARS-CoV2 Polymerase Chain Reaction (PCR) test | **Active infectious diseases such as active bacterial infections (defined as the isolation of bacteria from a sterile body site or other body sites and is causing clinical symptoms and signs and therefore are treated with antibiotics), invasive fungal infections, Human Immunodeficiency Virus (HIV), Hepatitis B Virus (HBV) infection, Hepatitis C Virus (HCV) infection, active tuberculosis** |
| Presence of respiratory distress:   - PaO2/FiO2 ≤ 300 mm Hg or - Respiratory Rate (RR) ≥24 breaths/min or - SpO2 ≤ 94% at room air) | **Patients on immunosuppressants or immunomodulatory drugs, or had received any in the past 30 days** |
| Signs of cytokine release syndrome as any of the following at baseline:   - Ferritin >600 mcg/L at presentation or >300 mcg/l with doubling within 24 hours, - LDH >250 IU/L, - D-dimers > 1 mg/L, - CRP > 70mg/L and rising since last 24h with the absence of bacterial infection, - Interleukin-6 level > 10 x UNL (reference range ≤7 pg/ml)], | **Neutrophil count below 500 cells/microliter,** |
| Radiological evidence of pneumonia based on chest X-ray and/or computed tomography (CT) scan findings | **Platelets below 50,000/microliter** |
| Signed informed consent provided by the patient, or by the patient's legal representative | **Pregnant or breastfeeding females.** |

**Supplementary Figure 2. Baseline characteristics of the participants**

| Characteristics | | Total | Anakinra Group | SOC Group | P-value | test used |
| --- | --- | --- | --- | --- | --- | --- |
|  |  |  |  |  |  |  |
| Hospital | n, % |  |  |  | 0.535 | Chi-square test |
|  | CDC | 18 (22.5) | 11 (27.5) | 7 (17.5) |  |  |
|  | HMGH | 44 (55.0) | 20 (50.0) | 24 (60.0) |  |  |
|  | TCH | 18 (22.5) | 9 (22.5) | 9 (22.5) |  |  |
| Nurse Unit | n, % |  |  |  | 1.000 | Chi-square test |
|  | General Ward | 70 (87.5) | 35 (87.5) | 35 (87.5) |  |  |
|  | ICU | 10 (12.5) | 5 (12.5) | 5 (12.5) |  |  |
| Age |  |  |  |  |  |  |
|  | mean (sd) | 49.9 (11.7) | 49.5 (12.2) | 50.3 (11.4) | 0.77 | Independent t-test |
| Gender | n, % |  |  |  | 0.556 | Chi-square test |
|  | Male | 66 (82.5) | 32 (80) | 34 (85) |  |  |
|  | Female | 14 (17.5) | 8 (20) | 6 (15) |  |  |
| WHO Nationality Region | n, % |  |  |  | 0.074 | Chi-square test |
|  | AFRO | 1 (1.3) | 1 (2.5) | 0 (0) |  |  |
|  | PAHO | 1 (1.3) | 0 (0) | 1 (2.5) |  |  |
|  | SEARO | 34 (42.5) | 13 (32.5) | 21 (52.5) |  |  |
|  | EURO | 2 (2.5) | 0 (0) | 2 (5.0) |  |  |
|  | EMRO | 26 (32.5) | 14 (35.0) | 12 (30.0) |  |  |
|  | WPRO | 16 (20.0) | 12 (30.0) | 4 (10.0) |  |  |
| BMI |  |  |  |  |  |  |
|  | median, IQR | 29.9 (27.3 - 32.9) | 30.9 (7.0) | 29.3 (5.2) | 0.285 | Mann-Whitney test |
| Smoking Status | n, % |  |  |  | 1.000 | Fisher's Exact test |
|  | Non-Smoker | 72 (90.0) | 36 (90.0) | 36 (90.0) |  |  |
|  | Current Smoker | 0 (0) | 0 | 0 |  |  |
|  | Ex-Smoker | 8 (10.0) | 4 (10.0) | 4 (10.0) |  |  |
| Has comorbidities | n, % |  |  |  | 0.501 | Chi-square test |
|  | Yes | 37 (46.3) | 17 (42.5) | 20 (50.0) |  |  |
|  | No | 43 (53.8) | 23 (57.5) | 20 (50.0) |  |  |
| List of comorbidities | n, % |  |  |  |  |  |
| YES | MI | 7 (8.8) | 2 (5.0) | 5 (12.5) | 0.432 | Fisher's Exact test |
|  | CHF | 0 |  |  | na |  |
|  | PVD | 0 |  |  | na |  |
|  | CVA_TIA | 0 |  |  | na |  |
|  | Dementia_cognitive Deficit | 0 |  |  | na |  |
|  | COPD | 1 (1.3) | 0 (0) | 1 (2.5) | 1.000 | Fisher's Exact test |
|  | Connective Tissue Disease | 0 |  |  | na |  |
|  | Peptic ulcer Disease | 0 |  |  |  |  |
|  | Liver Disease | 1 (1.3) | 1 (2.5) | 0 (0) | 1.000 | Fisher's Exact test |
|  | DM | 35 (43.8) | 17 (42.5) | 18 (45.0) | 0.822 | Chi-square test |
|  | Hemiplegia | 0 |  |  | na |  |
|  | Moderate to severe CKD | 1 (1.3) | 0 (0) | 1 (2.5) | 1.000 | Fisher's Exact test |
|  | Solid tumor | 0 |  |  |  |  |
|  | Leukemia | 0 |  |  |  |  |
|  | Lymphoma | 0 |  |  |  |  |
|  | AIDS | 0 |  |  |  |  |
| Charlson Comorbidity Index (CCI) | n, % |  |  |  |  |  |
|  | median (IQR) | 0.0 (0.0 - 1.0) | 0.0 (1) | 0.5 (1) | 0.425 | Mann-Whitney test |
|  | <2 | 72 (90.0) | 37 (92.5) | 35 (87.5) | 0.712 | Fisher's Exact test |
|  | ≥2 | 8 (10.0) | 3 (7.5) | 5 (12.5) |  |  |
| Symptomatic at baseline | n, % |  |  |  |  |  |
|  | Yes | 80 | 40 | 40 | na |  |
|  | No | 0 | 0 | 0 |  |  |
| Symptoms | n, % |  |  |  |  |  |
| YES | Cough | 75 (93.8) | 36 (90.0) | 39 (97.5) | 0.359 | Fisher's Exact test |
|  | Fever | 69 (86.3) | 34 (85.0) | 35 (87.5) | 0.745 | Chi-square test |
|  | SOB | 59 (73.8) | 29 (72.5) | 30 (75.0) | 0.799 | Chi-square test |
|  | Sore Throat | 12 (15.0) | 4 (10.0) | 8 (20.0) | 0.21 | Chi-square test |
|  | Loss of Taste/Smell | 5 (6.3) | 2 (5.0) | 3 (7.5) | 1.000 | Fisher's Exact test |
|  | Arthalgia_Myalgia_Body pain | 31 (38.8) | 15 (37.5) | 16 (40.0) | 0.818 | Chi-square test |
|  | Chest Pain | 15 (18.8) | 8 (20.0) | 7 (17.5) | 0.775 | Chi-square test |
|  | Headache_Dizziness | 13 (16.3) | 8 (20.0) | 5 (12.5) | 0.363 | Chi-square test |
|  | Fatigue_weakness | 14 (17.5) | 5 (12.5) | 9 (22.5) | 0.239 | Chi-square test |
|  | GI SE (N_V_D_abdominal pain) | 25 (31.3) | 9 (22.5) | 16 (40.0) | 0.091 | Chi-square test |
| Vitals (mean + sd) |  |  |  |  |  |  |
|  | Temp (Min) | 36.4 (0.2) | 36.32 (0.23) | 36.3 (0.41) | 0.148 | Independent t-test |
|  | Temp (Max) | 37.2 (0.6) | 37.1 (0.7) | 37.2 (0.6) | 0.971 | Independent t-test |
|  | SBP (Min) | 111.1 (13.0) | 119.2 (19.2) | 107.3 (16.9) | 0.585 | Independent t-test |
|  | SBP (Max) | 133.9 (15.5) | 162.0 (29.6) | 140.2 (15.4) | 0.416 | Independent t-test |
|  | DBP (Min) | 63.5 (8.9) | 73.2 (13.4) | 56.5 (7.3) | 0.7 | Independent t-test |
|  | DBP (Max) | 82.3 (10.4) | 101.8 (16.2) | 84.7 (9.5) | 0.717 | Independent t-test |
|  | HR (Min) | 67.2 (10.4) | 62.6 (7.4) | 67.5 (15.3) | 0.601 | Independent t-test |
|  | HR (Max) | 88.4 (13.4) | 95.4 (12.3) | 99.5 (33.9) | 0.185 | Independent t-test |
|  | RR (Min) | 18.1 (2.3) | 14.2 (5.4) | 18.5 (1.6) | 0.702 | Independent t-test |
|  | RR (Max) | 22.9 (4.4) | 29.2 (5.5) | 32.0 (4.9) | 0.781 | Independent t-test |
|  | O2 Sat % (Min) | 93.4 (2.4) | 93.0 (3.1) | 92.3 (2.7) | 0.782 | Independent t-test |
| Labs (mean (sd) /median (IQR)) |  |  |  |  |  |  |
|  | WBC | 6.7 (5.2-9.1) | 6.2 (4.5) | 8.1 (-) | 0.668 | Mann-Whitney test |
|  | Hgb | 13.1 (1.6) | 12.8 (1.6) | 14.3 (0.6) | 0.749 | Independent t-test |
|  | Platelets | 248 (200 - 300) | 211 (47) | 296 (-) | 0.637 | Mann-Whitney test |
|  | Neutrophils | 5.0 (3.8 - 7.1) | 4.2 (4.3) | 6.8 (-) | 0.637 | Mann-Whitney test |
|  | Lymphocytes | 1.1 (0.5) | 1.1 (0.6) | 0.8 (0.0) | 0.873 | Independent t-test |
|  | Eosinophils | 0.0 (0.0) | 0.0 (0.0) | 0.0 (0.0) | 0.181 | Independent t-test |
|  | D-Dimer | 0.6 (0.4 - 0.8) | 0.4 (0.5) | 0.5 (-) | 0.544 | Mann-Whitney test |
|  | Fibrinogen | 4.5 (4.0 - 7.0) | 4.3 (3.0) | 3.7 (-) | 0.918 | Mann-Whitney test |
|  | Urea | 5.4 (2.4) | 5.8 (3.4) | 7.4 (0.4) | 0.568 | Independent t-test |
|  | SCr | 86.0 (32.4) | 81.0 (20.0) | 83.5 (9.2) | 0.06 | Independent t-test |
|  | Na | 135.6 (3.6) | 137.3 (2.5) | 136.0 (1.4) | 0.474 | Independent t-test |
|  | K | 4.2 (0.5) | 4.4 (0.8) | 3.9 (0.2) | 0.178 | Independent t-test |
|  | BillirubinT | 9.1 (13.4) | 7.6 (3.5) | 7.8 (2.6) | 0.348 | Independent t-test |
|  | T_Protein | 69.6 (7.0) | 67.9 (6.6.) | 73.5 (5.0) | 0.238 | Independent t-test |
|  | Albumin | 31.8 (4.8) | 33.4 (7.1) | 32.8 (3.9) | 0.922 | Independent t-test |
|  | ALP | 71.3 (23.3) | 75.7 (28.4) | 73.0 (12.7) | 0.217 | Independent t-test |
|  | ALT | 42.9 (28.6) | 47.9 (21.6) | 70.5 (55.9) | 0.868 | Independent t-test |
|  | AST | 43.1 (20.0) | 45.3 (17.4) | 55.0 (41.0) | 0.937 | Independent t-test |
|  | LDH | 424.1 (130.4) | 450 (157.6) | 349.5 (108.2) | 0.988 | Independent t-test |
|  | CK | 298.3 (379.9) | 280.4 (292.6) | 195.5 (143.5) | 0.136 | Independent t-test |
|  | Lactic Acid | 3.9 (14.2) | 1.5 (0.4) | 1.7 (0.5) | 0.313 | Independent t-test |
|  | Glucose (mmol/L) | 9.6 (3.9) | 11.1 (5.8) | 11.9 (1.3) | 0.532 | Independent t-test |
|  | HbA1C % | 7.4 (1.9) | 6.8 (1.0) | 9.2 (1.0) | 0.796 | Independent t-test |
|  | CRP | 81.3 (55.5) | 41.0 (25.0) | 68.9 (26.7) | 0.768 | Independent t-test |
|  | Procalcitonin | 0.2 (0.3) | 0.2 (0.1) | 0.0 (0.0) | 0.714 | Independent t-test |
|  | Ferritin | 972.5 (695.1) | 1087.3 (568.2) | 990.5 (765.8) | 0.069 | Independent t-test |
|  | IL-6 | 23 (7 - 50) | 10.0 (9) | 5.5 (-) | 0.62 | Mann-Whitney test |
| On oxygen support at baseline | n, % |  |  |  |  |  |
|  | Yes | 80 (100) | 40 (100) | 40 (100) | na |  |
|  | No | 0 | 0 | 0 |  |  |
| WHO Score |  |  |  |  |  |  |
|  | median (IQR) | 5 (0) | 5.0 (0) | 5.0 (0) | 0.859 | Mann-Whitney test |
|  | 5 (%) | 69 (86.3) | 35 (87.5) | 34 (85.0) |  |  |
|  | 6 (%) | 8 (10.0) | 2 (5.0) | 6 (15.0) |  |  |
|  | 7 (%) | 3 (3.7) | 3 (7.5) | 0 |  |  |
| QuantiFERON | n, % |  |  |  | 0.658 | Chi-square test |
|  | Negative | 24 (30.4) | 12 (30.0) | 12 (30.0) |  |  |
|  | Indeterminate | 49 (62.0) | 26 (65.0) | 23 (57.5) |  |  |
|  | Positive | 6 (7.6) | 2 (5.0) | 4 (10.0) |  |  |
| COVID CT value |  |  |  |  |  |  |
|  | median (IQR) | 23.9 (19.7-27.2) | 23.9 (6.8) | 24.0 (8.6) | 0.988 | Mann-Whitney test |
| QTc value at baseline ECG |  |  |  |  |  |  |
|  | median (IQR) | 421.5 (403.3 - 441.8) | 423.5 (49) | 418.5 (45) | 0.15 | Mann-Whitney test |
| other ECG Findings | n, % |  |  |  | 0.125 | Chi-square test |
|  | NSR | 68 (85.0) | 34 (85.0) | 34 (85.0) |  |  |
|  | AF | 2 (2.5) | 1 (2.5) | 1 (2.5) |  |  |
|  | Bradycardia | 3 (3.8) | 3 (7.5) | 0 |  |  |
|  | Tachycardia | 4 (5.0) | 1 (2.5) | 3 (7.5) |  |  |
|  | Prolonged QT | na | na | na |  |  |
|  | Prolonged PR | 2 (2.5) | 0 | 2 (5.0) |  |  |
|  | RBBB, LBBB | 1 (1.2) | 1 (2.5) | 0 |  |  |
|  | No QRS Complex | na | na | na |  |  |
| Radiological Abnormalities | n, % |  |  |  |  |  |
| YES | Bilateral abnormality | 74 (92.5) | 38 (95.0) | 36 (90.0) | 0.675 | Fisher's Exact test |
|  | Ground-glass opacity | 22 (27.5) | 9 (22.5) | 13 (32.5) | 0.317 | Chi-square test |
|  | Consolidation | 23 (28.2) | 11 (27.5) | 12 (30.0) | 0.805 | Chi-square test |
|  | Infiltrates | 31 (38.8) | 18 (45.0) | 13 (32.5) | 0.251 | Chi-square test |
|  | Patchy Opacity | 48 (60.0) | 26 (65.0) | 22 (55.0) | 0.361 | Chi-square test |
| Medications |  |  |  |  |  |  |
|  | Anakinra (n, %) | 40 (100) | 40 (100) | 0 | 0 | Chi-square test |
|  | Total no. of doses (mean/sd) | 9.98 (0.16) | 9.98 (0.16) | na | na |  |
|  | remdesivir (n, %) | 67 (83.8) | 36 (90.0) | 31 (77.5) | 0.13 | Chi-square test |
|  | Rem duration (median, IQR) | 5 (5 - 5) | 5 (0) | 5 (0) | 0.255 | Mann-Whitney test |
|  | Favipiravir (n, %) | 42 (52.5) | 18 (45.0) | 24 (60.0) | 0.179 | Chi-square test |
|  | Favipiravir duration (median, IQR) | 4.0 (1.8 -7.0) | 3 (7) | 4.5 (5) | 0.718 | Mann-Whitney test |
|  | steroids (n, %) | 80 (100) | 40 (100) | 40 (100) | na |  |
|  | steroids duration (median, IQR) | 9.0 (7.3-10.0) | 10.0 (2) | 9.0 (3) | 0.603 | Mann-Whitney test |
|  | tocilizumab (n, %) | 4 (5.0) | 1 (2.5) | 3 (7.5) | 0.615 | Fisher's Exact test |
|  | no. of doses (median, IQR) | 1 (1-1) | na | 1 (0) | 1.000 | Mann-Whitney test |
|  | convalescent plasma (n, %) | 54 (67.5) | 24 (60.0) | 30 (75.0) | 0.152 | Chi-square test |
|  | Conv plasma duration | 2 (2 - 3) | 2 (1) | 2 (0) | 0.715 | Mann-Whitney test |
|  | ribavirin (n, %) | 1 (1.3) | 0 (0) | 1 (2.5) | 1.000 | Fisher's Exact test |
|  | Ribavirin duration | 1 (1 - 1) | na | na | na |  |
|  | interferon (n, %) | 1 (1.3) | 0 (0) | 1 (2.5) | 1.000 | Fisher's Exact test |
|  | Interferon duration | 1 (1 - 1) | na | na | na |  |
|  | Lopinavir /ritonavir (n, %) | 19 (23.8) | 7 (17.5) | 12 (30.0) | 0.189 | Chi-square test |
|  | Lopinavir /ritonavir duration | 1 (1 - 2) | 1 (1) | 1 (0) | 0.432 | Mann-Whitney test |
|  | Azithromycin /clarithromycin (n, %) | 63 (78.8) | 33 (82.5) | 30 (75.0) | 0.412 | Chi-square test |
|  | Azithromycin/  clarithromycin duration | 3 (2 - 3) | 3 (1) | 3 (0) | 0.253 | Mann-Whitney test |
|  | HCQ (n, %) | 11 (13.8) | 4 (10.0) | 7 (17.5) | 0.33 | Chi-square test |
|  | HCQ duration | 1 (1 - 2) | 1.5 (2) | 1 (0) | 0.527 | Mann-Whitney test |
|  | oseltamivir (n, %) | 2 (2.6) | 1 (2.5) | 1 (2.5) | 1.000 | Fisher's Exact test |
|  | oseltamivir duration | 1 (1 - 1) | na | na | na | Mann-Whitney test |
|  | ceftriaxone (n, %) | 63 (78.8) | 29 (72.5) | 34 (85.0) | 0.172 | Chi-square test |
|  | ceftriaxone duration | 7 (3 - 7) | 6 (5) | 7 (3) | 0.139 | Mann-Whitney test |
|  | Tazocin (n, %) | 15 (18.8) | 8 (20.0) | 7 (17.5) | 0.775 | Chi-square test |
|  | Tazocin duration | 7 (6 - 8) | 7 (3) | 6 (5) | 0.054 | Mann-Whitney test |
|  | Anticoagulant use ( (n, %) | 79 (98.8) | 39 (97.5) | 40 (100) | 1.000 | Fisher's Exact test |
|  | vasopressor use (n, %) | 3 (3.8) | 2 | 1 | 1.000 | Fisher's Exact test |

**Laboratory and vital signs results over time (2-way ANOVA Repeated measures over time)**

1. Vital signs


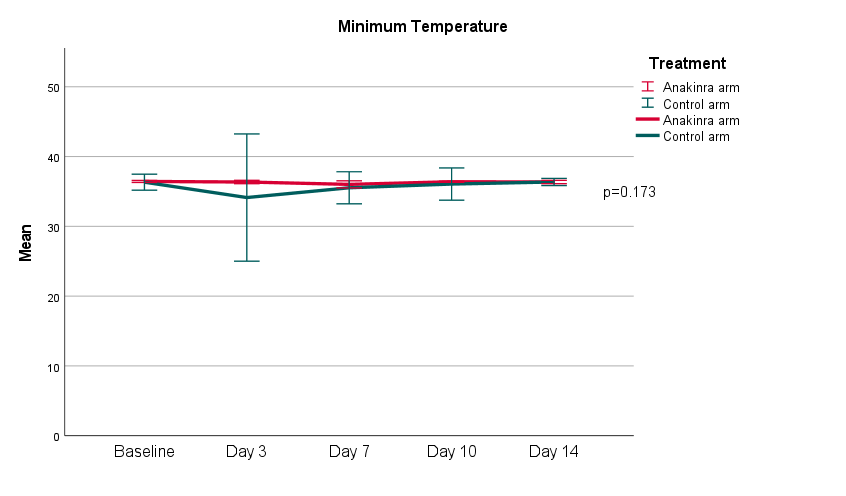


Supplementary Figure 1: Minimum Temperature over time


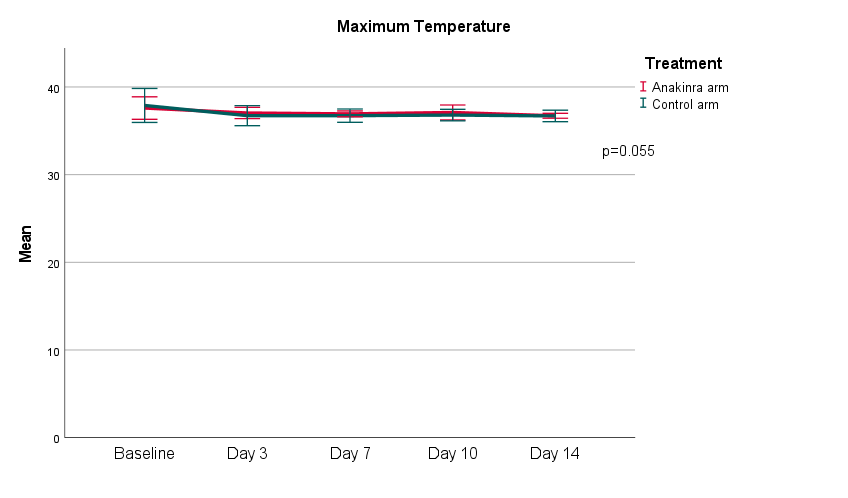


Supplementary Figure 2: Maximum temperature over time


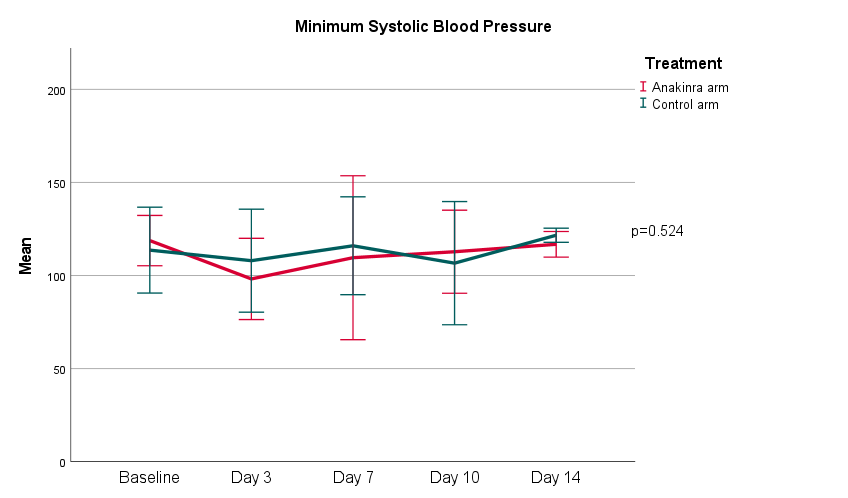


Supplementary Figure 3: Minimum systolic blood pressure over time


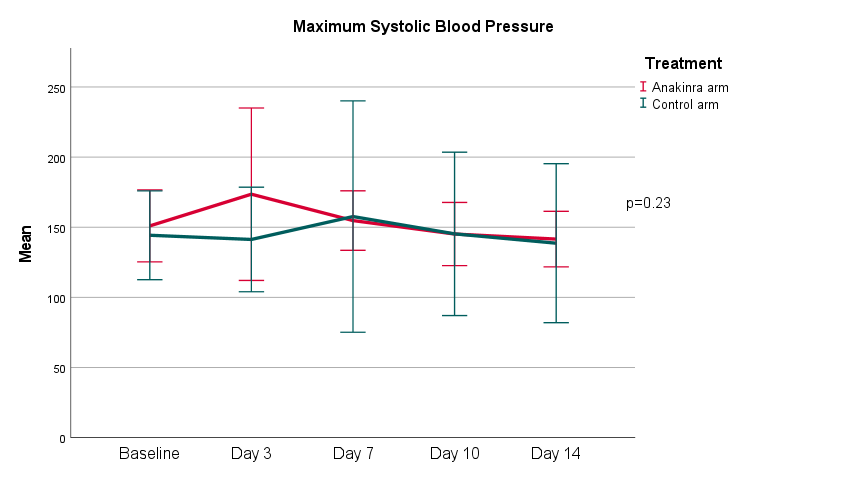


Supplementary Figure 4: Maximum systolic blood pressure over time


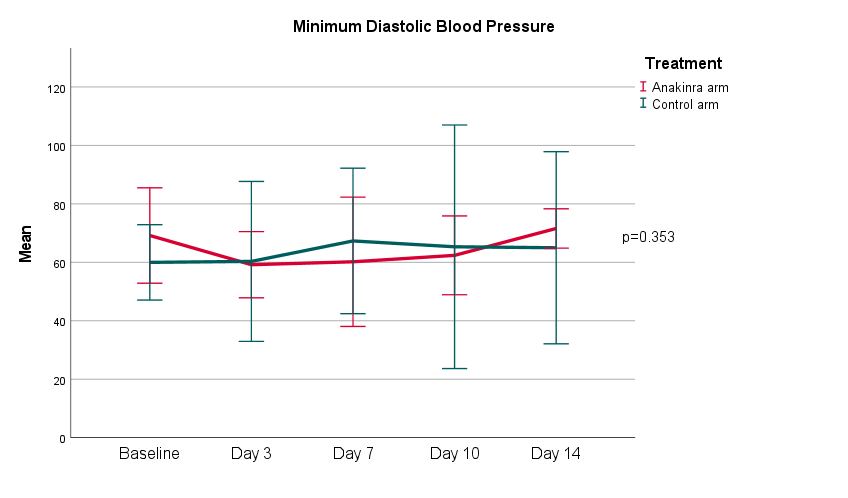


Supplementary Figure 5: Minimum diastolic blood pressure over time


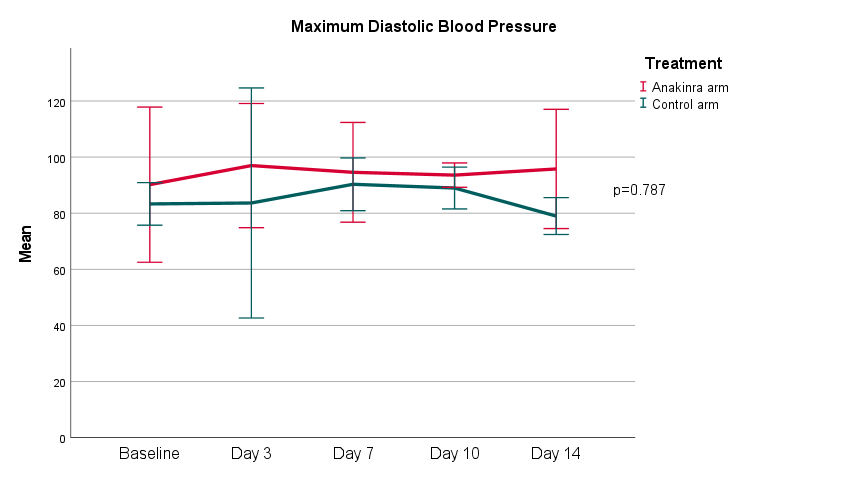


Supplementary Figure 6: Maximum diastolic blood pressure over time


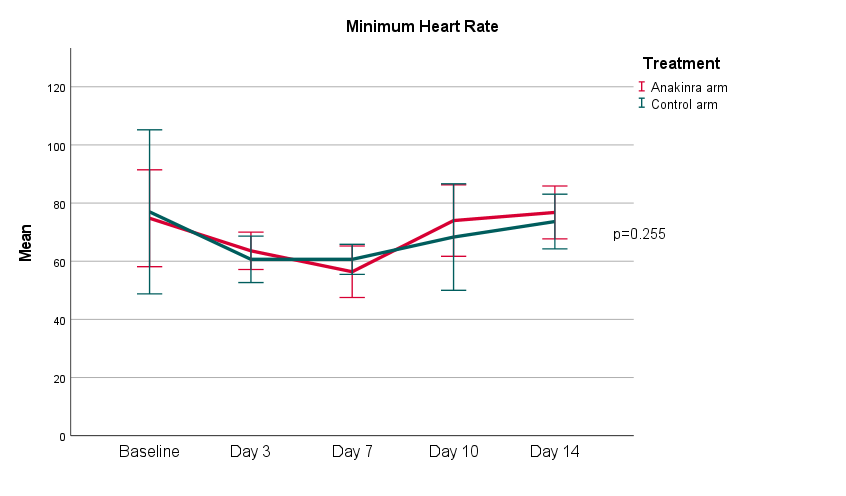


Supplementary Figure 7: Minimum heart rate over time


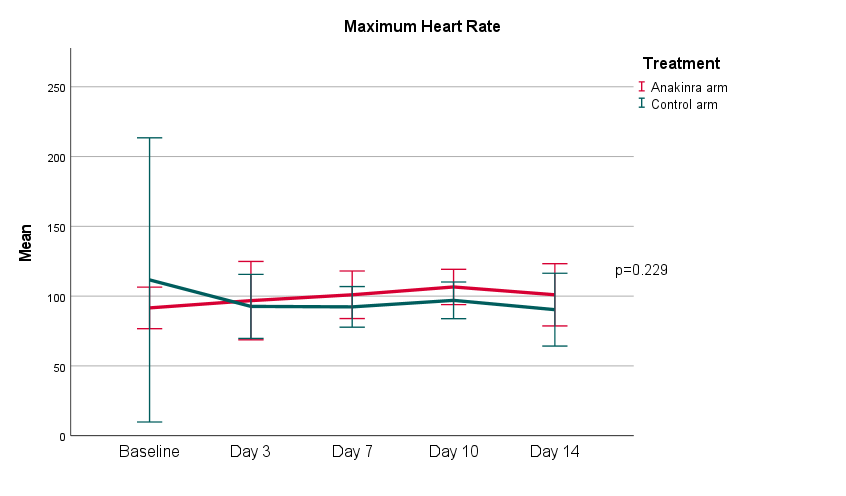


Supplementary Figure 8: Maximum heart rate over time


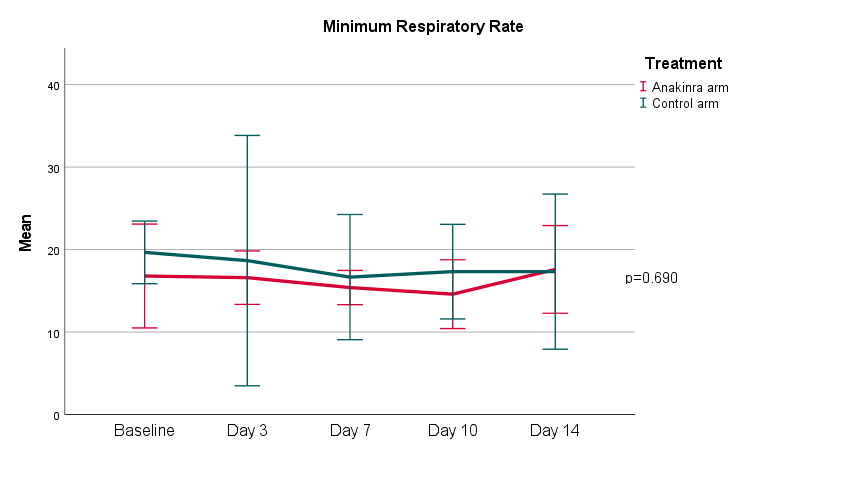


Supplementary Figure 9: Minimum respiratory rate over time


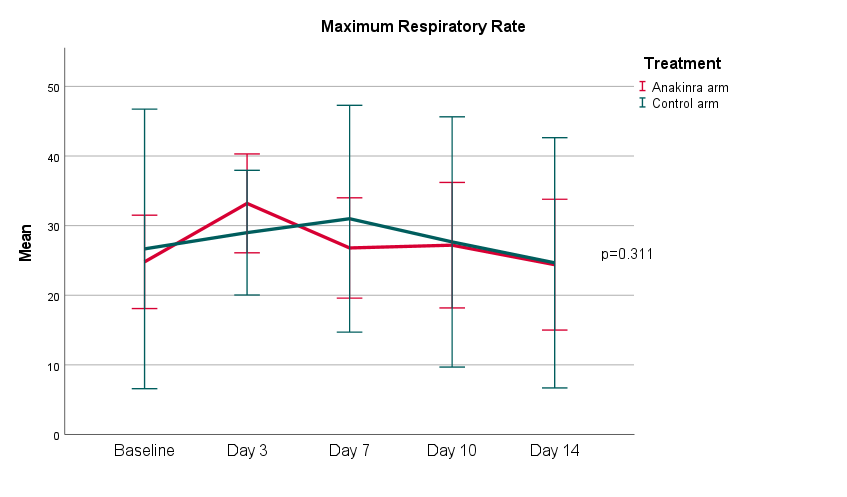


Supplementary Figure 10: Maximum respiratory rate over time


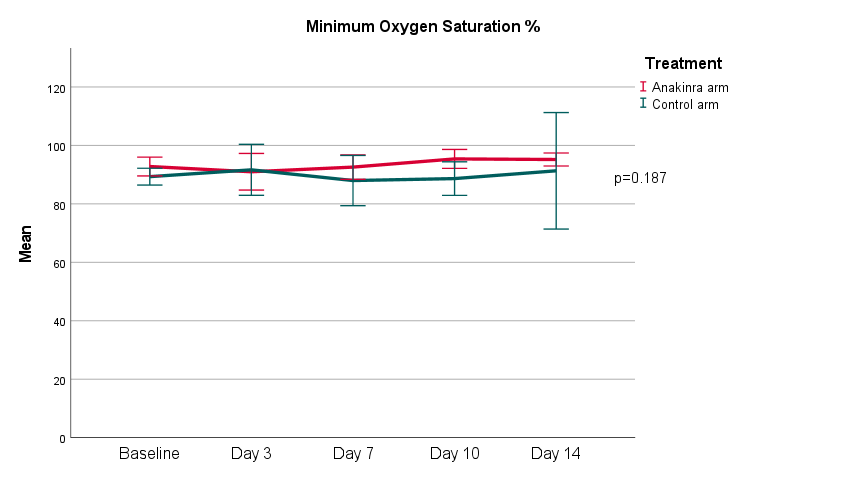


Supplementary Figure 11: Maximum oxygen saturation over time

1. Lab data


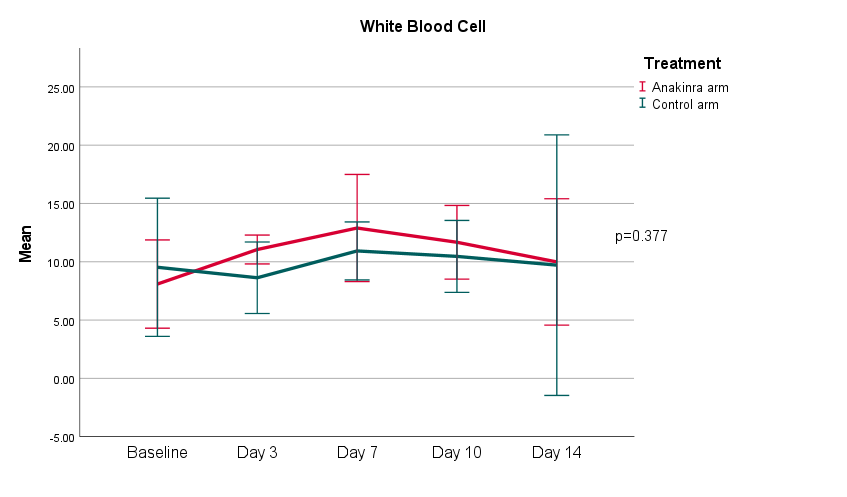


Supplementary Figure 12: White blood cell count over time


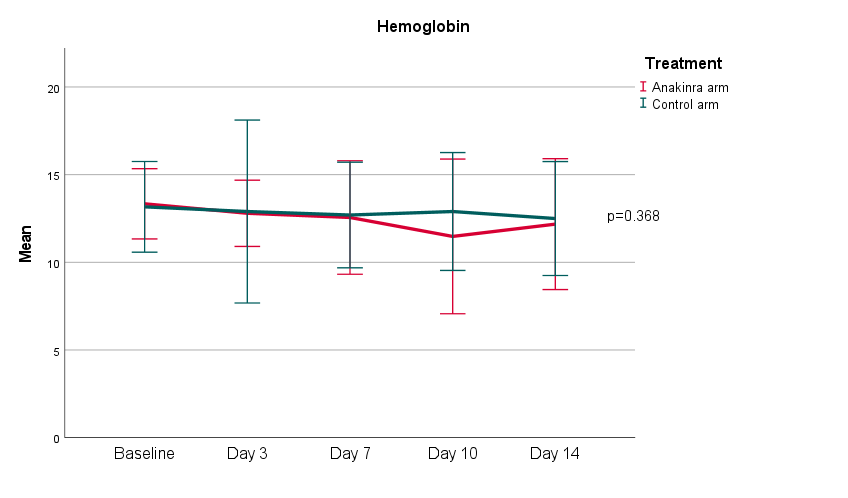


Supplementary Figure 13: Hemoglobin count over time


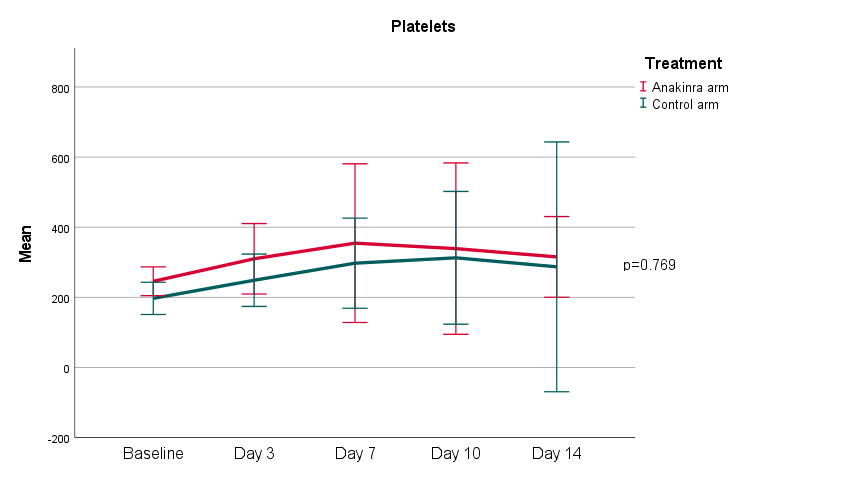


Supplementary Figure 14: Platelets count over time


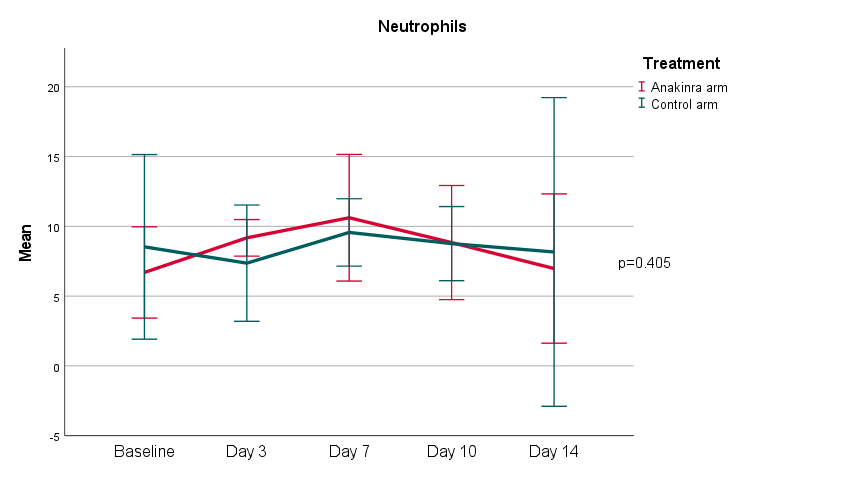


Supplementary Figure 15: Neutrophils count over time


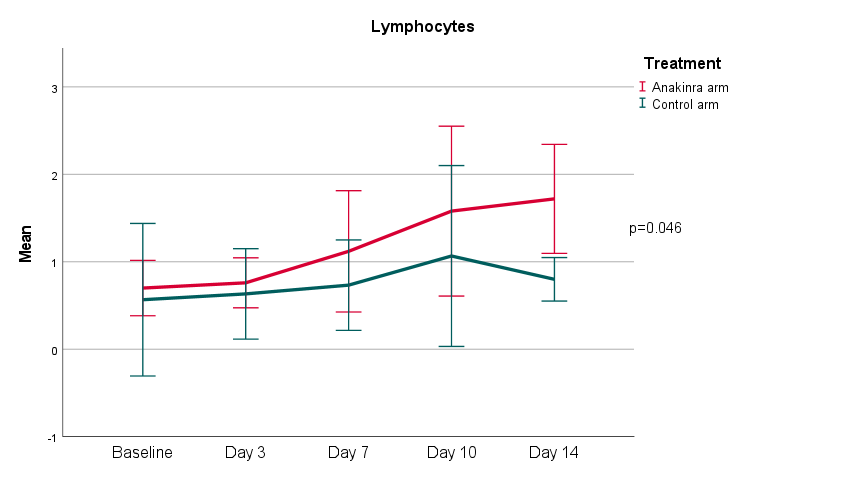


Supplementary Figure 16: Lymphocytes count over time


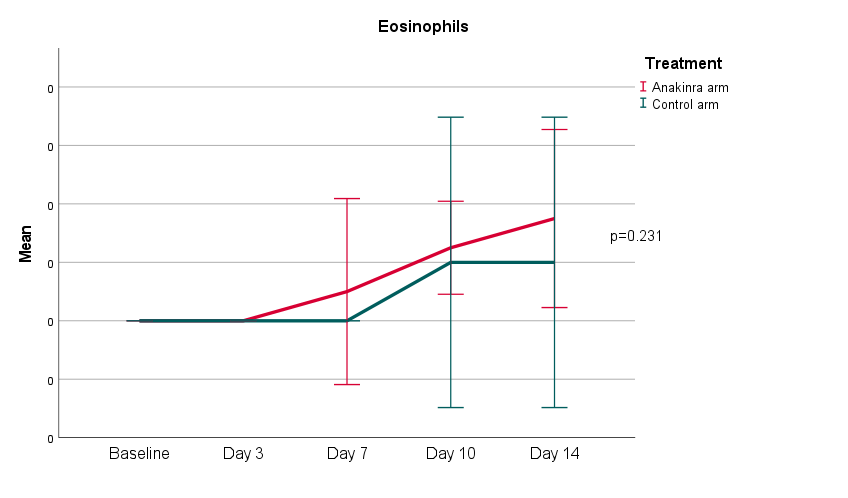


Supplementary Figure 17: Eosinophils count over time


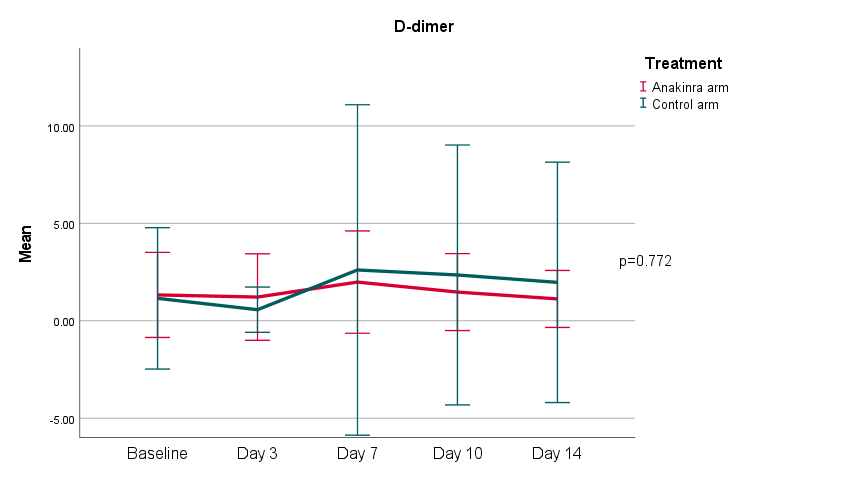


Supplementary Figure 18: D-dimer level over time


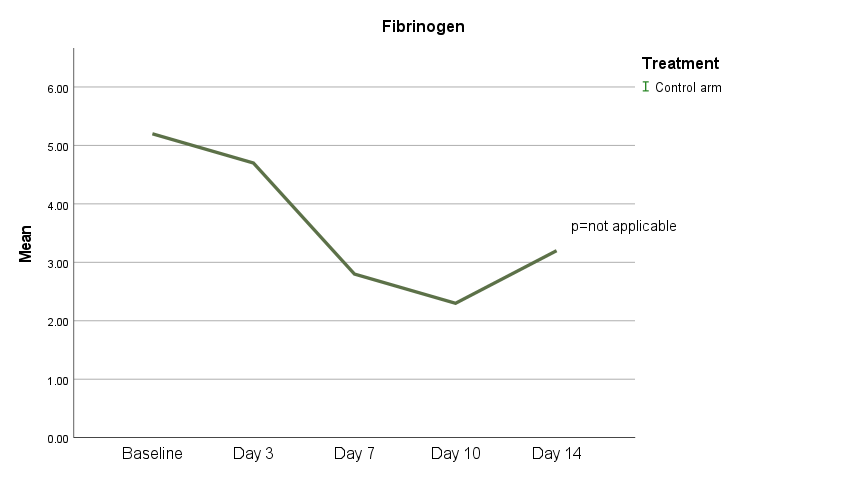


Supplementary Figure 19: Fibrinogen level over time


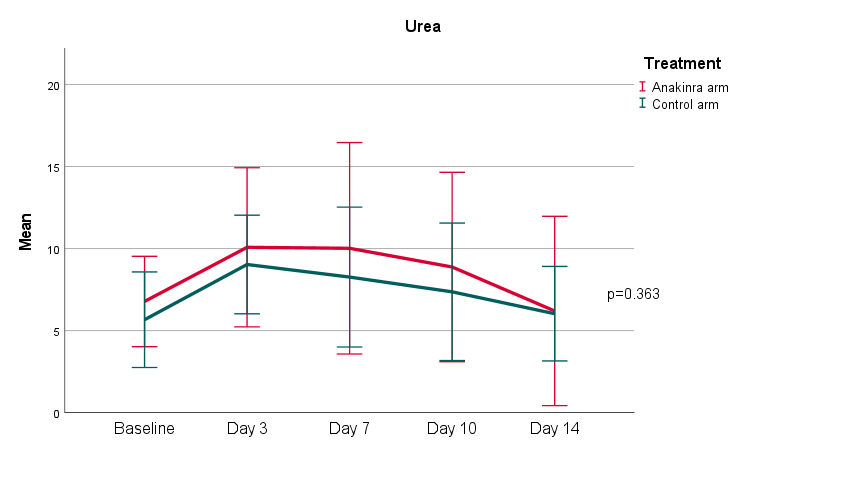


Supplementary Figure 20: Urea level over time


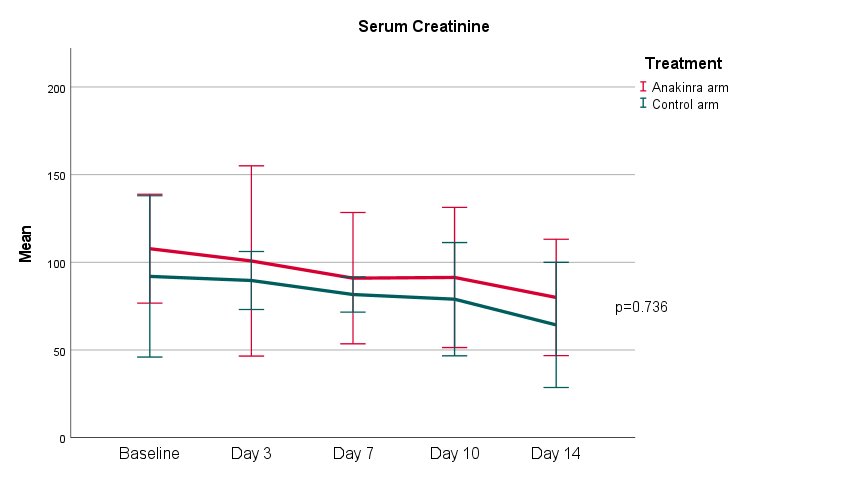


Supplementary Figure 21: Serum creatinine level over time


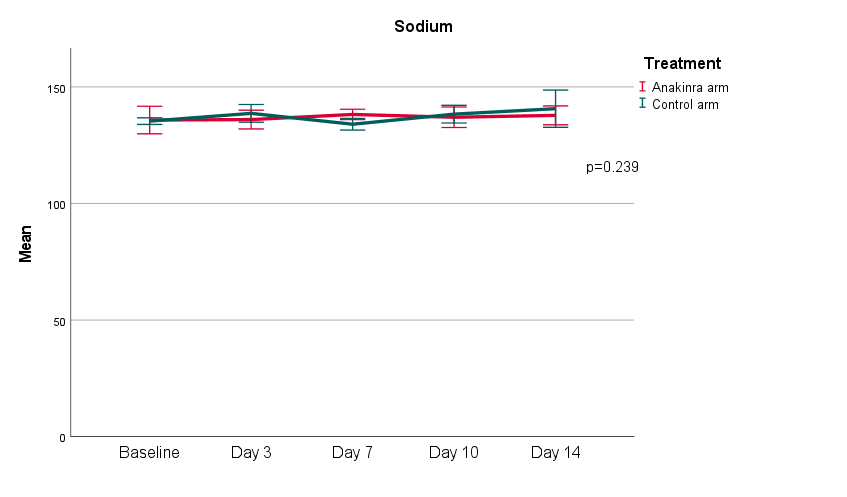


Supplementary Figure 22: Sodium level over time


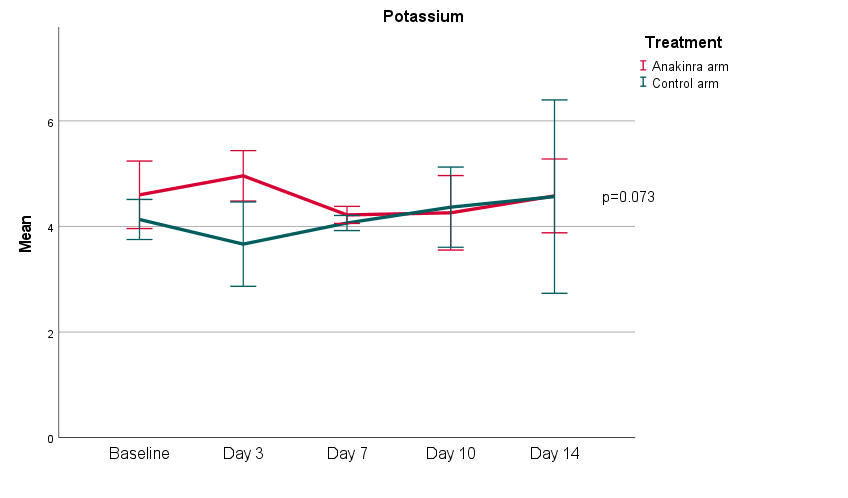


Supplementary Figure 23: Potassium level over time


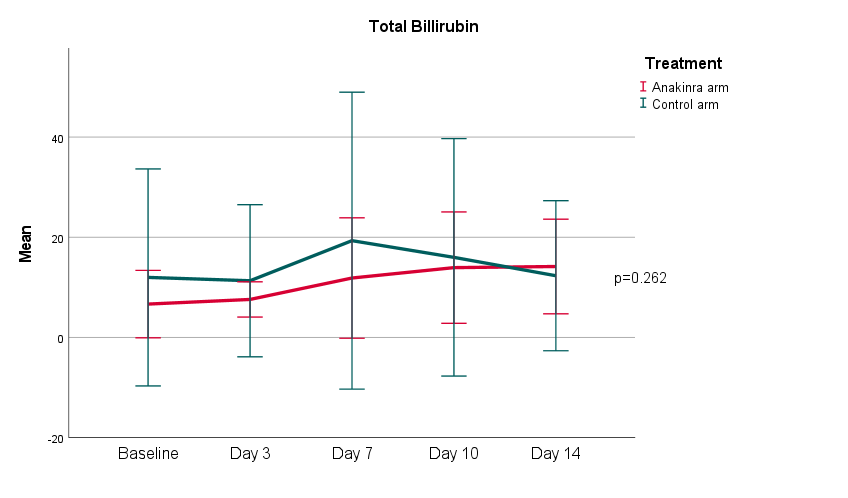


Supplementary Figure 24: Total bilirubin level over time


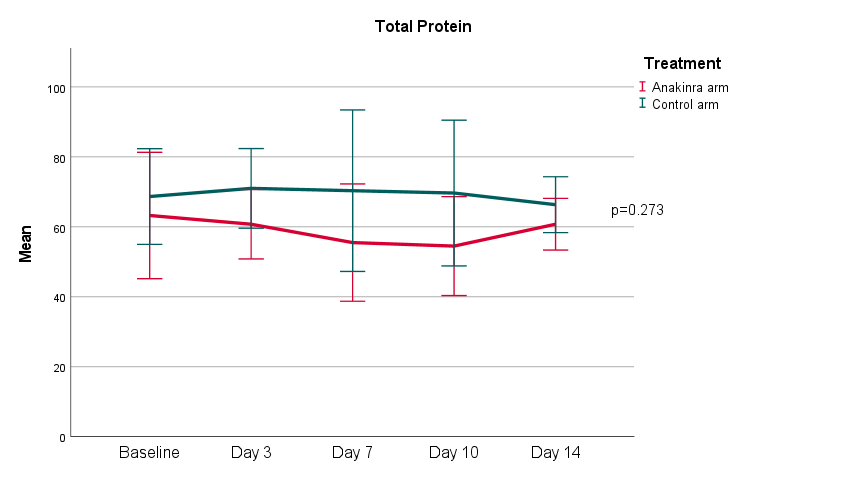


Supplementary Figure 25:Total protein level over time


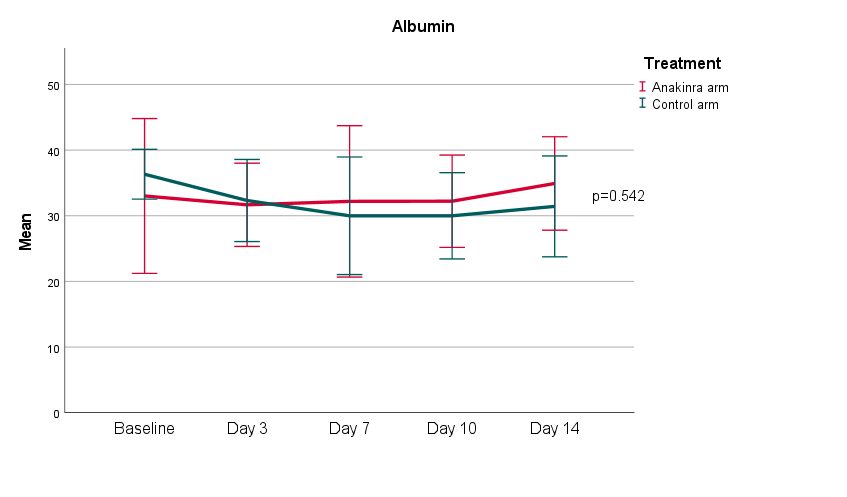


Supplementary Figure 26: Albumin level over time


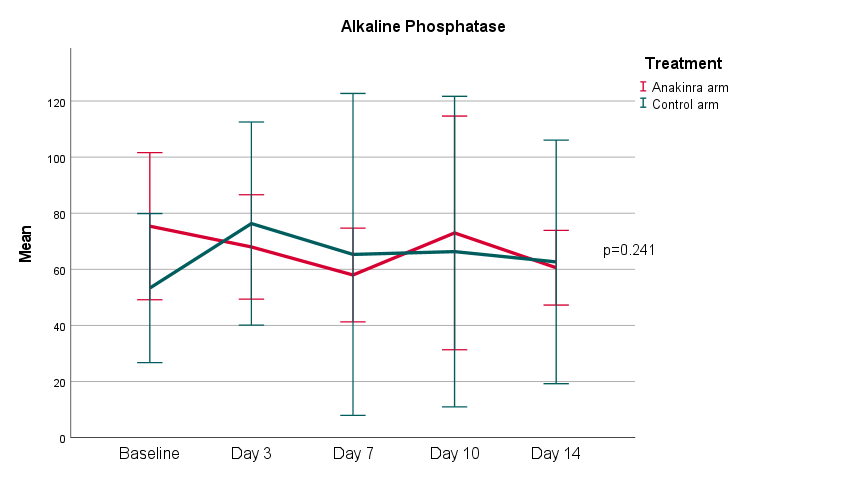


Supplementary Figure 27: Alkaline phosphatase level over time


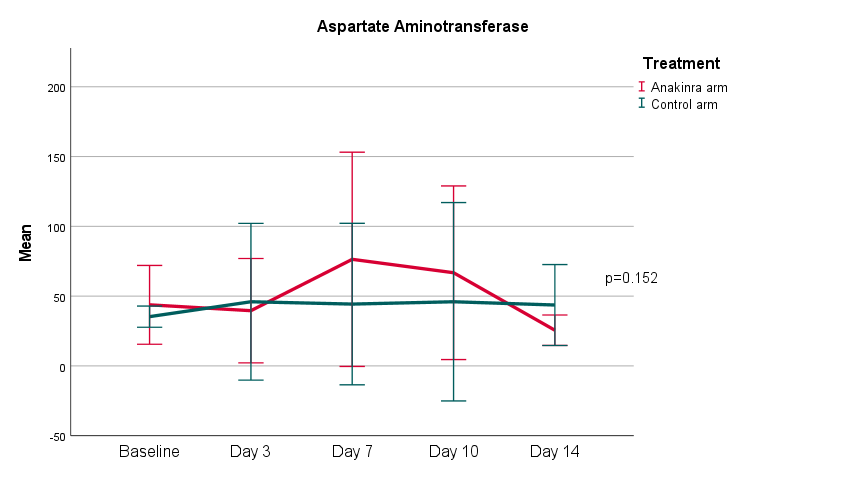


Supplementary Figure 28: Aspartate Aminotransferase level over time


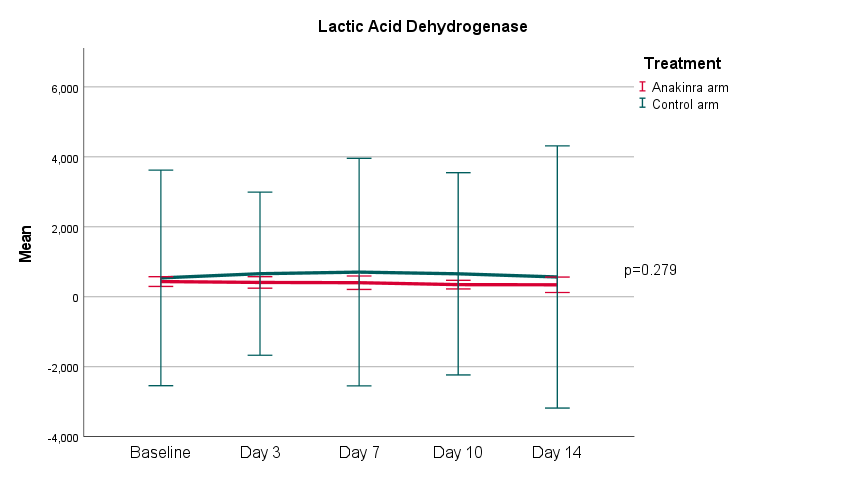


Supplementary Figure 29: Lactic acid dehydrogenase level over time


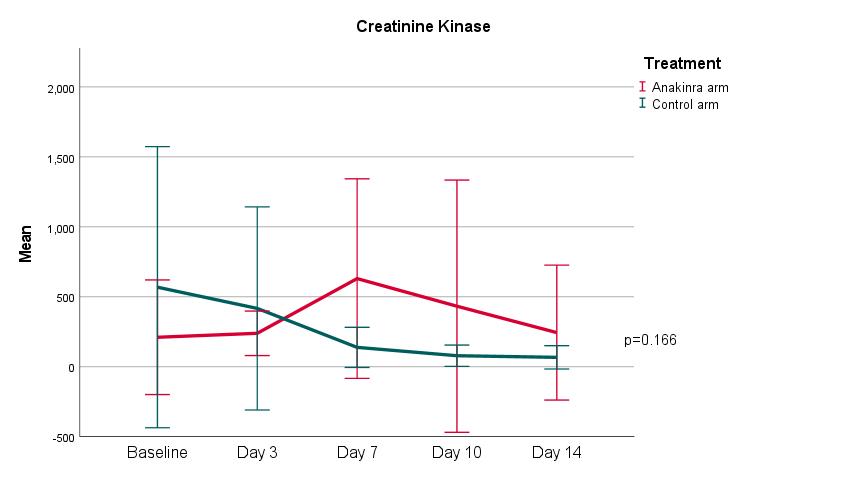


Supplementary Figure 30: Creatinine kinase level over time


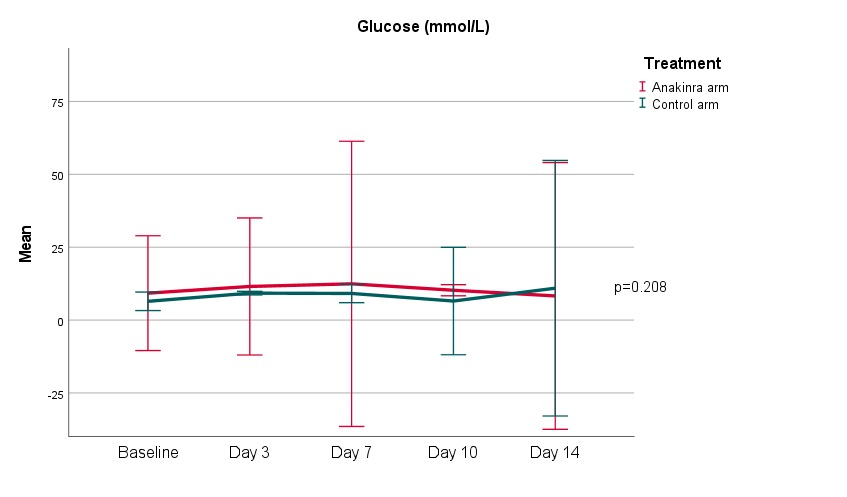


Supplementary Figure 31: Glucose level over time


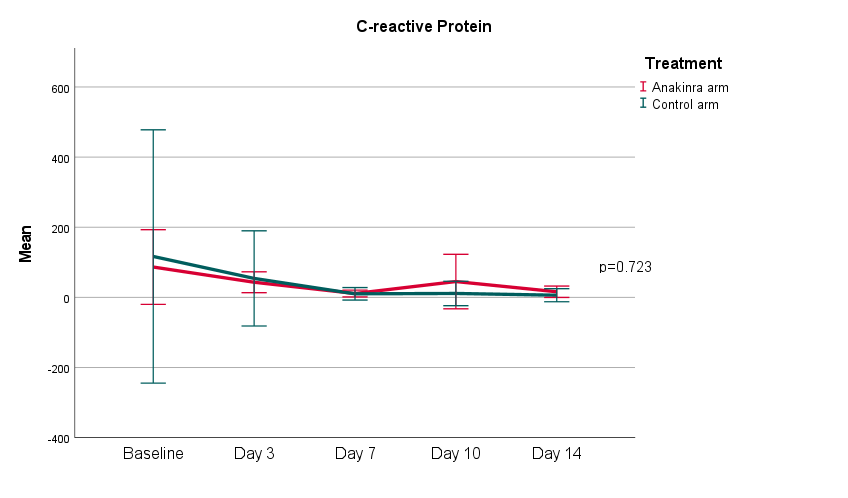


Supplementary Figure 32: C-reactive protein level over time


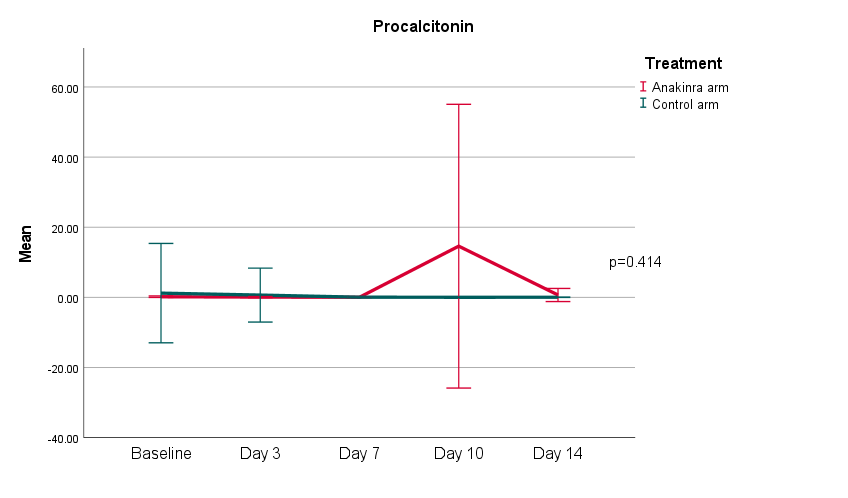


Supplementary Figure 33: Procalcitonin level over time


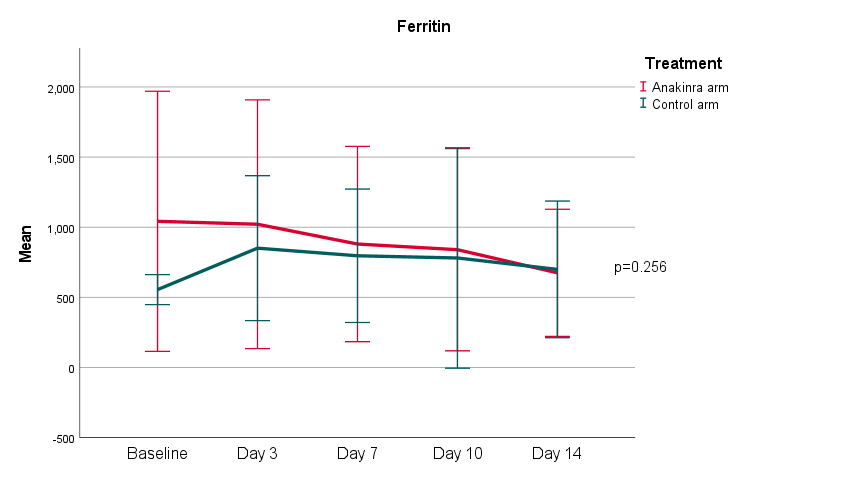


Supplementary Figure 34: Ferritin level over time


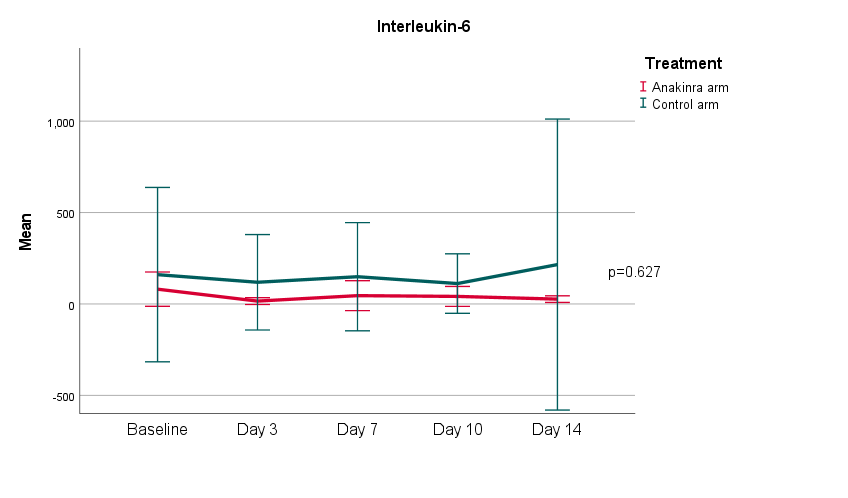


Supplementary Figure 35: Interleukin-6 level over time


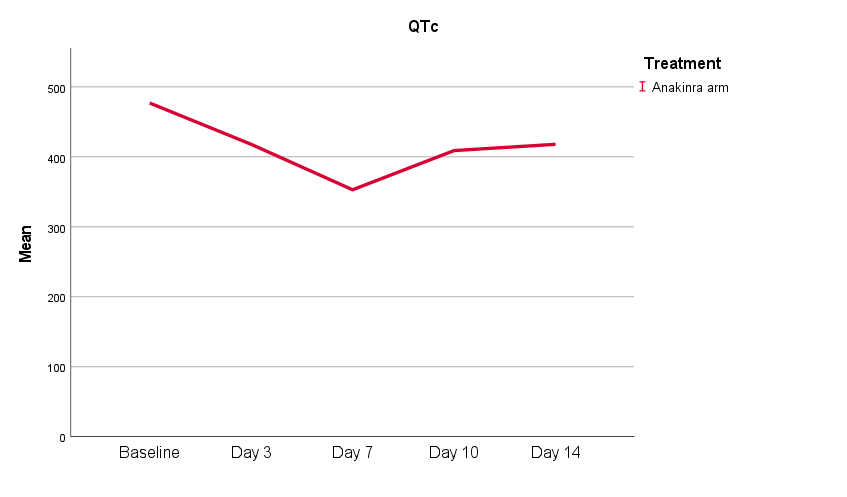


Supplementary Figure 36: QTc interval over time


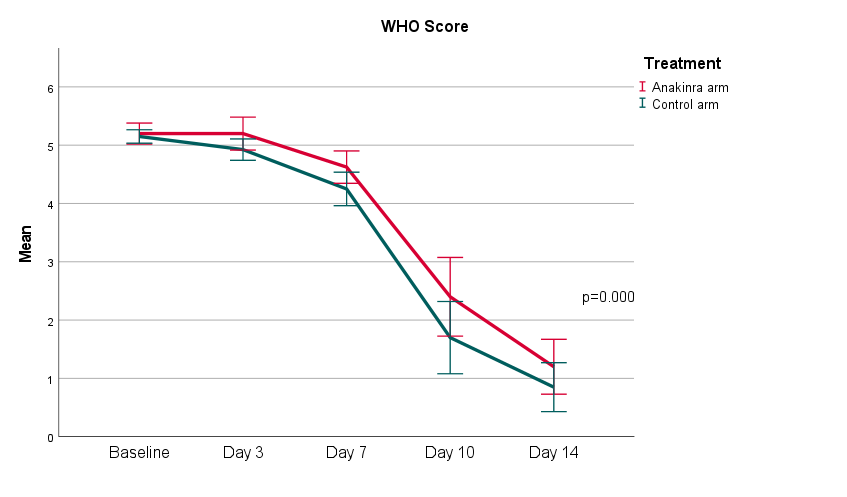


Supplementary Figure 37: WHO Score over time

**Supplementary Table 3. Association between baseline characteristics and primary outcome**

|  |  | Total | Treatment Group A | Treatment Group B | P-value | test used |
| --- | --- | --- | --- | --- | --- | --- |
| Percentage of patients with treatment success at day 14 | |  | Yes | Yes |  |  |
|  | Gender | Male | 27 (84.4) | 31 (91.2) | 0.469 | Fisher's exact test |
|  |  | Female | 8 (100) | 6 (100) | 0.712 | Fisher's exact test |
|  |  |  |  |  |  |  |
|  | Nationality (Region)AFRO |  | 1 (100) | na | na |  |
|  |  | EMRO | 13 (92.9) | 10 (83.3) | 0.58 | Fisher's exact test |
|  |  | EURO | na | 2 (100) | na |  |
|  |  | PAHO | na | 1 (100) | na |  |
|  |  | SEARO | 11 (84.6) | 20 (95.2) | 0.544 | Fisher's exact test |
|  |  | WPRO | 10 (83.3) | 4 (100) | 1.000 | Fisher's exact test |
|  |  |  |  |  |  |  |
|  | Hospital | CDC | 10 (90.9) | 6 (85.7) | 1.000 | Fisher's exact test |
|  |  | HMGH | 19 (95.0) | 22 (91.7) | 1.000 | Fisher's exact test |
|  |  | TCH | 6 (100) | 9 (100) | 0.206 | Fisher's exact test |
|  |  |  |  |  |  |  |
|  | Nursing unit | ICU | 3 (60) | 4 (80) | 1.000 | Fisher's exact test |
|  |  | Ward | 32 (91.4) | 33 (94.3) | 1.000 | Fisher's exact test |
|  |  |  |  |  |  |  |
|  | Smoking | 0 | 32 (88.9) | 34 (94.4) | 0.674 | Fisher's exact test |
|  |  | 2 | 3 (75.0) | 3 (75.0) | 1.000 | Fisher's exact test |
|  |  |  |  |  |  |  |
|  | Age |  | 48.9 (12.1) | 48.9 (10.5) | 0.143 | 2-way ANOVA |
|  | BMI |  | 30.5 (5.5) | 30.4 (5.2) | 0.287 | 2-way ANOVA |
|  |  |  |  |  |  |  |
|  | Has comorbidities | No | 21 (60) | 19 (51.4) | 0.46 | Chi-Square test |
|  |  | Yes | 14 (40) | 18 (48.6) |  |  |
|  | MI | No | 33 (94.3) | 34 (91.9) | 0.69 | Chi-Square test |
|  |  | Yes | 2 (5.7) | 3 (8.1) |  |  |
|  | DM | No | 21 (60.0) | 20 (54.1) | 0.576 | Chi-Square test |
|  |  | Yes | -40 | 17 (45.9) |  |  |
|  | SOB | No | 11 (31.4) | 9 (24.3) | 0.501 | Chi-Square test |
|  |  | Yes | 24 (68.6) | 28 (75.7) |  |  |
|  | Baseline WHO Score |  | 5.2 (0.6) | 5.2 (0.4) | 0.015 | 2-way ANOVA |
|  |  |  |  |  |  |  |
|  | Bilateral abnormalities (x-ray) | No | 2 (5.7) | 4 (10.8) | 0.434 | Chi-Square test |
|  |  | Yes | 33 (94.3) | 33 (89.2) |  |  |
|  | Remdesivir | No | 4 (11.4) | 9 (24.3) | 0.155 | Chi-Square test |
|  |  | Yes | 31 (88.6) | 28 (75.7) |  |  |
|  | Tocilizumab | No | 35 (100) | 36 (97.3) | 0.327 | Chi-Square test |
|  |  | Yes | 0 | 1 (2.7) |  |  |
|  | Convalescent plasma | No | 16 (45.7) | 10 (27.0) | 0.099 | Chi-Square test |
|  |  | Yes | 19 (54.3) | 27 (73.0) |  |  |

**Supplementary Table 4. Completed list of adverse drug event for the trial population**

| Adverse events, n (%) | Total | Group A | Group B | P-value | Test used |
| --- | --- | --- | --- | --- | --- |
| Abdominal pain | 1 (1.3) | 1 (2.5) | 0 (0) | 1.000 | Fisher's Exact test |
| Alanine aminotransferase increased | 35 (43.8) | 19 (47.5) | 16 (40.0) | 0.499 | Chi-square test |
| Alkaline phosphatase increased | 8 (10.0) | 5 (12.5) | 3 (7.5) | 0.712 | Fisher's Exact test |
| Anemia | 21 (26.3) | 8 (20.0) | 13 (32.5) | 0.204 | Chi-square test |
| Arthralgia | 1 (1.3) | 1 (2.5) | 0 (0) | 1.000 | Fisher's Exact test |
| Aspartate aminotransferase increased | 20 (25.0) | 14 (35.0) | 6 (15.0) | 0.039 | Chi-square test |
| Atrial Fibrilliation | 1 (1.3) | 1 (2.5) | 0 (0) | 1.000 | Fisher's Exact test |
| Blood bilirubin increased | 3 (3.9) | 1 (2.5) | 2 (5.0) | 1.000 | Fisher's Exact test |
| Bronchial infection | 1 (1.3) | 1 (2.5) | 0 (0) | 1.000 | Fisher's Exact test |
| Chest pain | 1 (1.3) | 0 (0) | 1 (2.5) | 1.000 | Fisher's Exact test |
| Constipation | 3 (3.9) | 2 (5.0) | 1 (2.5) | 1.000 | Fisher's Exact test |
| CPK Increased | 2 (2.6) | 0 (0) | 2 (5.0) | 0.494 | Fisher's Exact test |
| Creatinine increased | 4 (5.2) | 2 (5.0) | 2 (5.0) | 1.000 | Fisher's Exact test |
| Dehydration | 2 (2.6) | 0 (0) | 2 (5.0) | 0.494 | Fisher's Exact test |
| Diarrhea | 8 (10.0) | 3 (7.5) | 5 (12.5) | 0.712 | Fisher's Exact test |
| ECG QT corrected interval prolonged | 9 (11.3) | 5 (12.5) | 4 (10.0) | 1.000 | Fisher's Exact test |
| Fever | 1 (1.3) | 1 (2.5) | 0 (0) | 1.000 | Fisher's Exact test |
| Flank pain | 2 (2.6) | 2 (5.0) | 0 (0) | 0.494 | Fisher's Exact test |
| Hallucinations | 1 (1.3) | 0 (0) | 1 (2.5) | 1.000 | Fisher's Exact test |
| Headache | 2 (2.6) | 0 (0) | 2 (5.0) | 0.494 | Fisher's Exact test |
| Hematoma | 1 (1.3) | 1 (2.5) | 0 (0) | 1.000 | Fisher's Exact test |
| Hemorrhoids | 1 (1.3) | 1 (2.5) | 0 (0) | 1.000 | Fisher's Exact test |
| Hyperglycemia | 17 (21.3) | 9 (22.5) | 8 (20.0) | 1.000 | Fisher's Exact test |
| Hyperkalemia | 7 (8.8) | 5 (12.5) | 2 (5.0) | 0.432 | Fisher's Exact test |
| Hypertension | 52 (65.0) | 27 (67.5) | 25 (62.5) | 0.639 | Chi-square test |
| Hypoalbuminemia | 35 (43.8) | 18 (45.0) | 17 (42.5) | 0.822 | Chi-square test |
| Hypoglycemia | 2 (2.6) | 1 (2.5) | 1 (2.5) | 1.000 | Fisher's Exact test |
| Hypokalemia | 7 (8.8) | 3 (7.5) | 4 (10.0) | 1.000 | Fisher's Exact test |
| Hyponatremia | 20 (25.0) | 11 (27.5) | 9 (22.5) | 0.606 | Chi-square test |
| Hypotension | 32 (40.0) | 19 (47.5) | 13 (32.5) | 0.171 | Chi-square test |
| Hypothermia | 2 (2.6) | 0 (0) | 2 (5.0) | 0.494 | Fisher's Exact test |
| Injection site reaction | 2 (2.6) | 2 (5.0) | 0 (0) | 0.494 | Fisher's Exact test |
| Lung infection | 2 (2.6) | 2 (5.0) | 0 (0) | 0.494 | Fisher's Exact test |
| Lymphocyte count decreased | 15 (18.8) | 8 (20.0) | 7 (17.5) | 0.775 | Chi-square test |
| Lymphocyte count increased | 4 (5.2) | 2 (5.0) | 2 (5.0) | 1.000 | Fisher's Exact test |
| Myalgia | 2 (2.6) | 2 (5.0) | 0 (0) | 0.494 | Fisher's Exact test |
| Pain in extremity | 1 (1.3) | 1 (2.5) | 0 (0) | 1.000 | Fisher's Exact test |
| Rash | 3 (3.9) | 1 (2.5) | 2 (5.0) | 1.000 | Fisher's Exact test |
| Sinus Bradycardia | 35 (43.8) | 16 (40.0) | 19 (47.5) | 0.499 | Chi-square test |
| Sinus tachycardia | 6 (21.7) | 4 (10.0) | 2 (5.0) | 0.675 | Fisher's Exact test |
| Stomach Pain | 1 (1.3) | 0 (0) | 1 (2.5) | 1.000 | Fisher's Exact test |
| Thromboembolic event | 3 (3.9) | 2 (5.0) | 1 (2.5) | 1.000 | Fisher's Exact test |
| Urticaria | 1(1.3) | 0 (0) | 1 (2.5) | 1.000 | Fisher's Exact test |
